# Estimate of the sequenced proportion of the global prokaryotic genome

**DOI:** 10.1186/s40168-020-00903-z

**Published:** 2020-09-16

**Authors:** Zheng Zhang, Jianing Wang, Jinlan Wang, Jingjing Wang, Yuezhong Li

**Affiliations:** 1 State Key Laboratory of Microbial Technology, Institute of Microbial Technology, Shandong University, Qingdao, 266237 China; 2 Suzhou Research Institute, Shandong University, Suzhou, 215123 China; 3 Physical Examination Office of Shandong Province, Health Commission of Shandong Province, Jinan, 250014 China

**Keywords:** Microbiome, Genome sequencing, Prokaryotic biome, Earth microbiome project, Predominant taxa

## Abstract

**Background:**

Sequencing prokaryotic genomes has revolutionized our understanding of the many roles played by microorganisms. However, the cell and taxon proportions of genome-sequenced bacteria or archaea on earth remain unknown. This study aimed to explore this basic question using large-scale alignment between the sequences released by the Earth Microbiome Project and 155,810 prokaryotic genomes from public databases.

**Results:**

Our results showed that the median proportions of the genome-sequenced cells and taxa (at 100% identities in the 16S-V4 region) in different biomes reached 38.1% (16.4–86.3%) and 18.8% (9.1–52.6%), respectively. The sequenced proportions of the prokaryotic genomes in biomes were significantly negatively correlated with the alpha diversity indices, and the proportions sequenced in host-associated biomes were significantly higher than those in free-living biomes. Due to a set of cosmopolitan OTUs that are found in multiple samples and preferentially sequenced, only 2.1% of the global prokaryotic taxa are represented by sequenced genomes. Most of the biomes were occupied by a few predominant taxa with a high relative abundance and much higher genome-sequenced proportions than numerous rare taxa.

**Conclusions:**

These results reveal the current situation of prokaryotic genome sequencing for earth biomes, provide a more reasonable and efficient exploration of prokaryotic genomes, and promote our understanding of microbial ecological functions.

Video Abstract

## Background

Prokaryotes are generally assumed to be the oldest existing form of life on earth and the primary engines of global biogeochemical processes; they are found in almost all ecosystems [1, 2]. Genome sequencing provides a blueprint for the evolutionary and functional diversities of prokaryotes and improves our understanding of how they interact with one another, their hosts, and their surroundings [3–5]. However, what is the cells or taxa proportion of genome-sequenced bacteria or archaea on earth? This basic and seemingly simple question has never been answered.

Since the first bacterial genome was completely sequenced in 1995, more than 200,000 bacterial and archaeal complete or draft genomes have been uploaded to public databases as a result of the development of sequencing technology and the decrease in costs [6, 7]. Meanwhile, due to improvements in sequencing throughput and computational techniques, cultivation-independent recovery of genomes from metagenomes further promotes prokaryotic genome mining [8–10]. Interestingly, compared to the exponential accumulation of genomic data, the latest estimate of global prokaryotic operational taxonomic units (OTUs, 16S-V4 regions at 97% sequence identities) is only 0.8–1.6 million, far less than the trillions previously predicted [11, 12]. It is necessary to globally evaluate the proportion of sequenced prokaryotic genomes in environments.

The Earth Microbiome Project (EMP) was founded in 2010 to sample and explore the Earth’s microbial communities at an unprecedented scale [13–15]. In this study, we conducted a large-scale sequence alignment between the data released by the EMP and the sequenced bacterial or archaeal genomes in the public database. From these data, we evaluated the present situation of prokaryotic genome sequencing in the earth biomes for the first time.

## Results

### High genome-sequenced proportions in different prokaryotic biomes

**Fig. 1:**
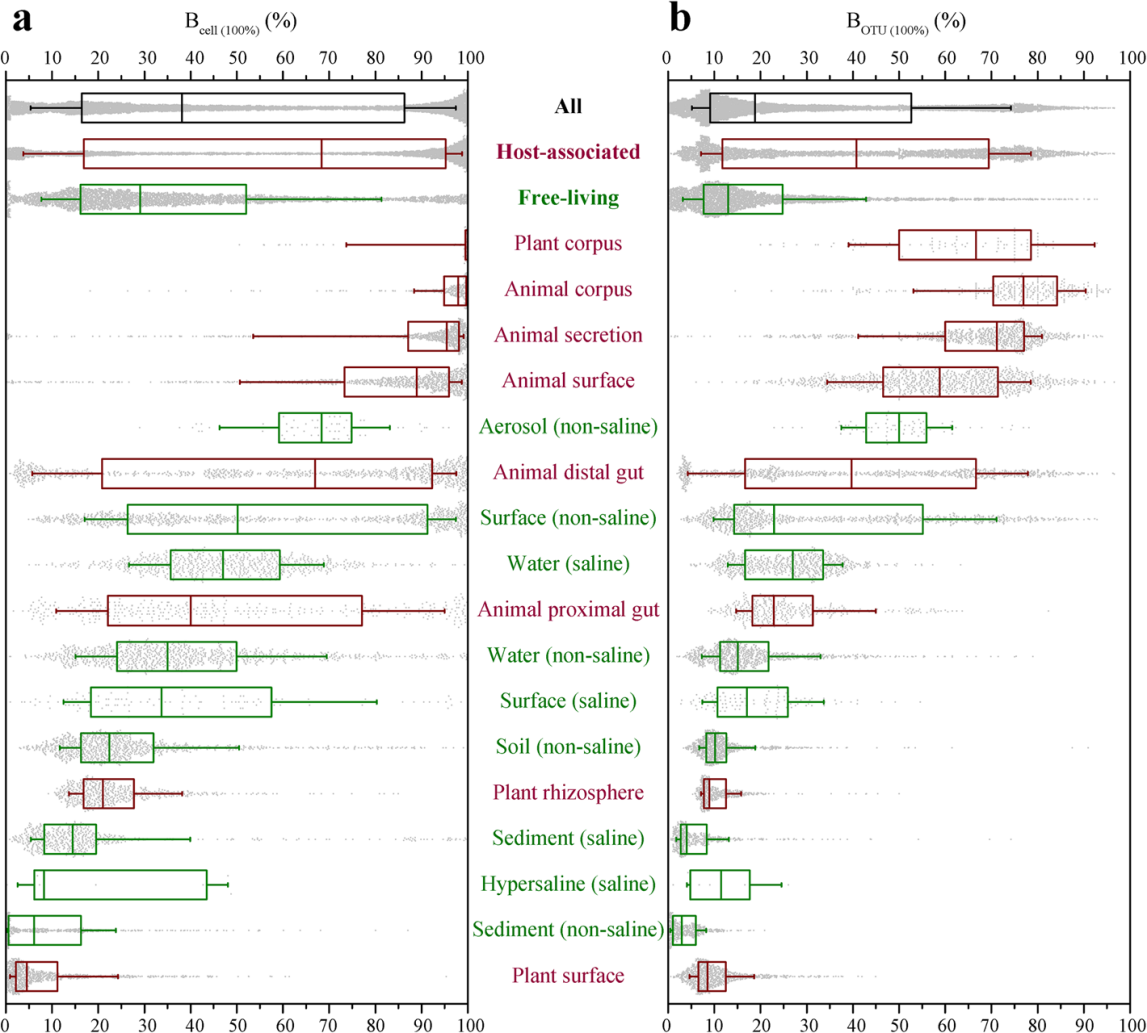
Genome-sequenced degree of prokaryotic biomes. **a** Genome-sequenced proportion of cells. **b** Genome-sequenced proportion of taxa. OTUs share 100% identities with the sequenced genomes. Based on the analysis of 10,000 EMP samples, each gray point represents a single sample. For the box plots, the middle line indicates the median, the box represents the 25th–75th percentiles, and the error bar indicates the 10th–90th percentiles of observations. Environment types were classified by EMPO; red represents host associated and green represents free living

A representative subset, containing 10,000 samples to represent different environment types, was selected from 27,751 samples of 97 independent studies released by the EMP [13]. *B*_cell_ and *B*_OTU_, which represent the genome-sequenced proportions of cells and taxa (at 100%, > 98.6%, or > 97% identities in the 16S-V4 region) in a specific prokaryotic biome, respectively, were evaluated based on the alignment between the 16S rRNA gene sequences of the EMP and the nearly 155,810 RefSeq genome sequences. The results showed that the median *B*_cell (100%)_ in the 10,000 samples was 38.1%, and the upper and lower quartiles were 16.4% and 86.3%, respectively (Fig. 1a and Supplementary dataset 1). This finding indicates that the genome information of at least 38% of cells has been reported in more than half of the prokaryotic biomes. The median *B*_OTU (100%)_ reached 18.8% (9.1–52.6%) (Fig. 1b). Generally, closely related strains with high similarities of 16S rRNA gene sequences (97% or 98.6%) also share high genome similarity [16–18]. The median *B*_cell (98.6%)_ was 50.1% (28.3–90.6%) whereas the median *B*_cell (97%)_ reached 60.4% (40.0–93.0%) across the 10,000 samples. Similarly, the median *B*_OTU (98.6%)_ was 28.4% (16.4–64.3%), and the median *B*_OTU (97%)_ increased to 37.6% (24.8–71.4%) (Supplementary Figs. S1, S2).

The genome-sequenced proportion in the prokaryotic biome was closely related to habitat (Fig. 1 and Supplementary dataset 2). Microbial environments are divided into different environment types by the EMP. The EMP ontology (EMPO level 1) classifies microbial environments as free living and host associated, with further subdivision into 17 environment types (EMPO level 3) [13]. We found that the genome-sequenced proportions in host-associated biomes were significantly higher than those in free-living biomes. For the host-associated prokaryotic biomes (5161 samples), the median *B*_cell (100%)_ was as high as 68.3% (16.9–95.2%), and the median *B*_OTU (100%)_ was 40.7% (11.8–69.4%). However, for the free-living prokaryotic biomes (4839 samples), the median *B*_cell (100%)_ was only 29.1% (16.2–52.0%), and the median *B*_OTU (100%)_ was 13.0% (7.7–24.8%). In detail, the median *B*_cell (100%)_ in plant corpus, animal corpus, and animal secretions exceeded 95%, and the median *B*_OTU (100%)_ exceeded 66.7%. Comparatively, the median *B*_cell (100%)_ values for plant surface, sediment (non-saline), and hypersaline samples were all less than 10%, and the median *B*_OTU (100%)_ values for sediment (non-saline) and sediment (saline) samples were less than 5% (Fig. 1). For closely related strains, *B*_cell_ and *B*_OTU_ also showed similar variabilities among different habitats (Supplementary Figs. S1, S2). Despite significant differences, the genome-sequenced proportions were high in most of the prokaryotic biomes.

Furthermore, we found that the genome-sequenced proportion in the prokaryotic biome was significantly negatively correlated with its alpha diversity indices (Supplementary Fig. S3). For both cells and taxa, the prokaryotic biomes with low alpha diversity indices (observed OTUs, Shannon index, Chao1 index, and Faith’s PD value) tended to have a higher degree of genome sequencing. For example, the Pearson correlation coefficients of *B*_cell (100%)_ and *B*_OTU (100%)_ with Shannon indices were − 0.62 (*p* < 0.01) and − 0.67 (*p* < 0.01), respectively.

### Low genome-sequenced proportions of global prokaryotic taxa

**Fig. 2:**
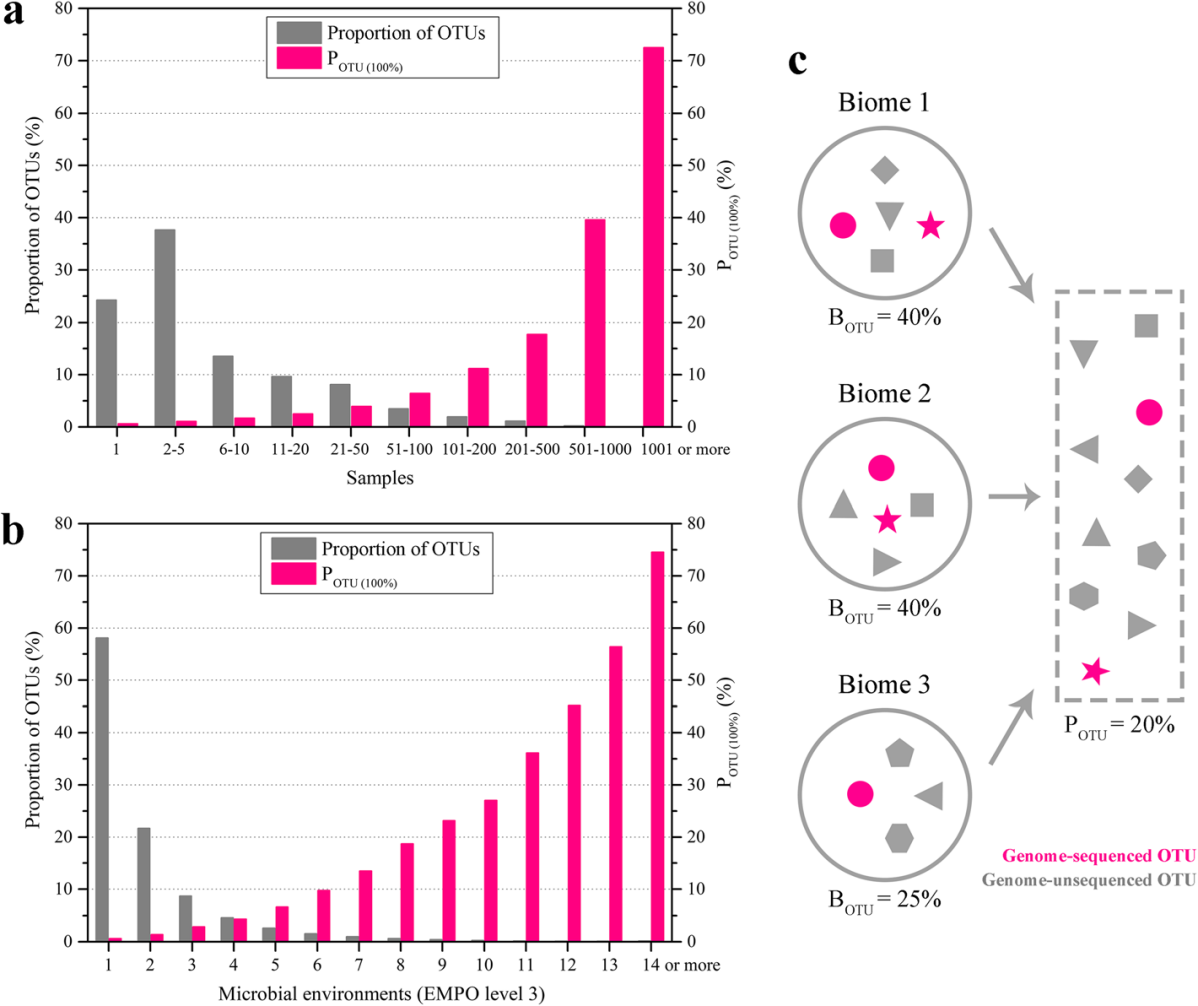
High genome-sequenced proportion of prokaryotic cosmopolitan taxa. **a** OTUs that can exist in one or more samples. **b** OTUs that can exist in one or more environment types. The gray column represents the proportion of OTUs that can exist in one or more samples (environments), and the red column represents the genome-sequenced proportion of OTUs. **c** Lower *P*_OTU_ than *B*_OTU_ is caused by a high genome-sequenced proportion of cosmopolitan taxa

A total of 262,011 OTUs were obtained from 10,000 EMP samples through a meta-analysis. We defined the genome-sequenced proportion of all taxa (at 100%, > 98.6%, or > 97% identities in the 16S-V4 region) as *P*_OTU_ and found that the *P*_OTU (100%)_ of the 10,000 samples was only 2.1% (Supplementary dataset 3). The *P*_OTU (98.6%)_ and *P*_OTU (97%)_ values were 6.8% and 12.2%, respectively, and both were also much lower than the corresponding *B*_cell_ and *B*_OTU_ medians. Furthermore, we found that 75.8% of OTUs were present in two or more biome samples. The *P*_OTU (100%)_ value was 0.6% for the OTUs that appeared in only a single sample (401 of 63,459 OTUs), 1.2% for those in 2 to 10 samples (1641 of 134,119 OTUs), 5.4% for those in more than 10 samples (3478 of 64,433 OTUs), 16.2% for those in more than 100 samples (1431 of 8810 OTUs), and 72.5% for those in more than 1000 samples (108 of 149 OTUs) (Fig. 2a). Notably, many prokaryotic taxa could exist in diverse environment types; approximately 21.7% of prokaryotic taxa could exist in two types of environments, and 20.2% of OTUs could exist in three or more types of environments. We found that the taxon genome-sequenced proportion also increased with its distribution extent in different environment types. The *P*_OTU (100%)_ was only 0.6% for prokaryotic OTUs that existed in only one type of environment (932 of 152,229 OTUs), 14.5% for OTUs in five or more types of environments (2645 of 18,230 OTUs), 43.6% for 10 or more types of environments (904 of 2074 OTUs), and 74.6% for 14 or more types of environments (287 of 385 OTUs) (Fig. 2b). A higher genome-sequenced proportion of prokaryotic cosmopolitan OTUs led to a lower *P*_OTU_ than the corresponding *B*_OTU_ (Fig. 2c).

**Fig. 3:**
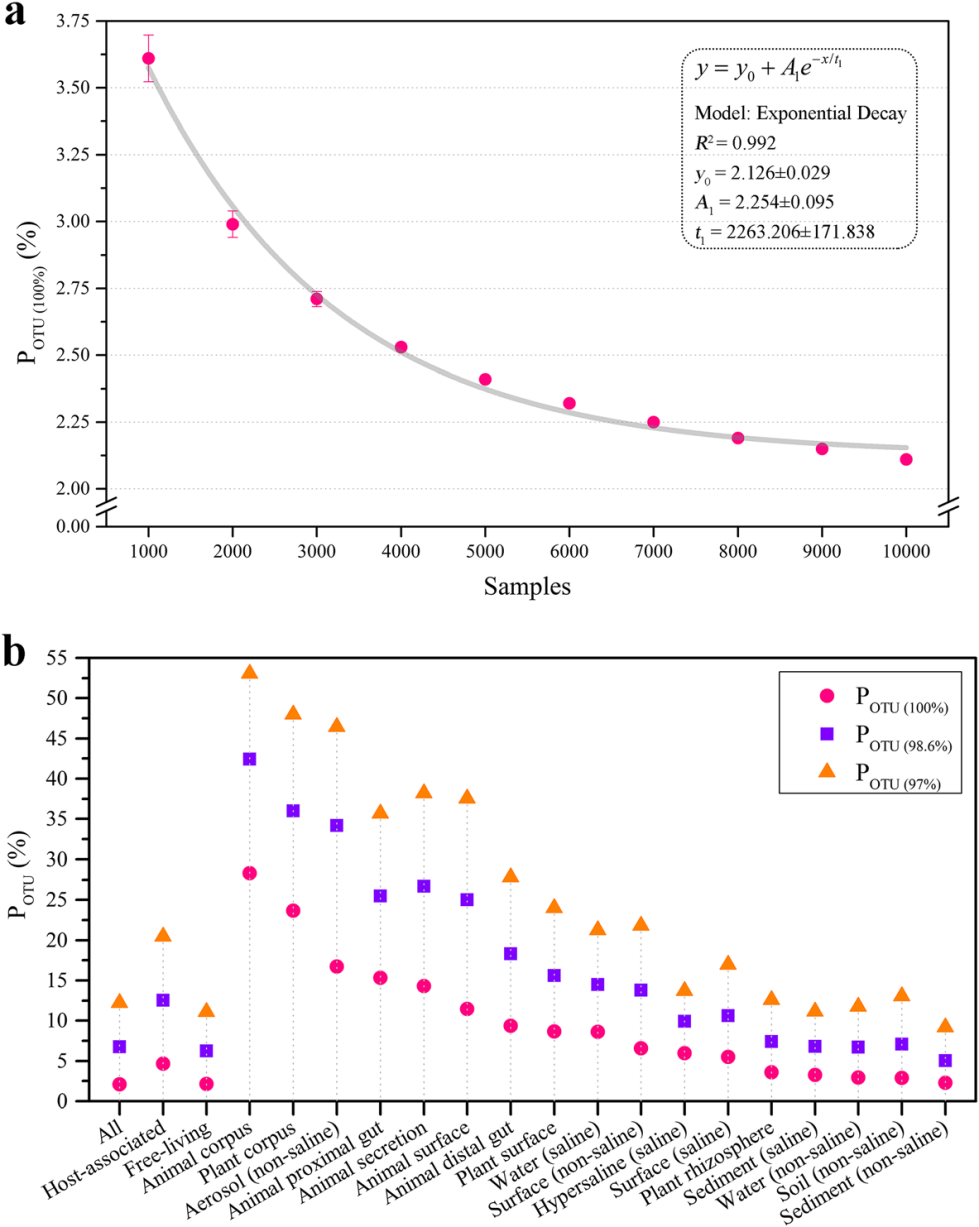
Genome-sequenced proportion of prokaryotic taxa from global or different environment types. **a** As the number of samples increases, the *P*_OTU (100%)_ shows an exponential declining trend and finally stabilizes at 2.1%. A random selection of 1000, 2000…, 9000 samples was performed 10 times for each group to calculate the mean value and standard deviation. **b** Significant difference of *P*_OTU_ among environment types. The red point is *P*_OTU (100%)_, the blue point is *P*_OTU (98.6%)_, and the orange point is *P*_OTU (97%)_

Because an OTU was likely to appear in multiple samples, we evaluated the effects of sample quantity on *P*_OTU_ by random sampling. Our results demonstrated that the *P*_OTU (100%)_ displayed an exponential decay trend (*R*^2^ = 0.992) and eventually stabilized at 2.13% ± 0.03% as the number of samples increased (Fig. 3a). Similarly, the *P*_OTU (98.6%)_ and *P*_OTU (97%)_ values also decreased with increasing sample size and stabilized at approximately 6.8% and 12.2%, respectively (Supplementary Fig. S4). The estimated *P*_OTU_ values based on 10,000 EMP samples were close to the genome-sequenced proportions in all global prokaryotic taxa. We evaluated the changes in *P*_OTU_ as the number of sequenced genomes increased from 2010 to 2019. The results showed that the *P*_OTU (100%)_ increased exponentially (*R*^2^ = 0.998) by sixfold over the decade. However, it was estimated that it would take at least 25 years for the *P*_OTU (100%)_ to reach 95%. With the increase in sequenced genomes, the *P*_OTU (100%)_ value showed an allometric increase (*R*^2^ = 0.989), and we determined that the 95% *P*_OTU (100%)_ value required more than 10^9^ sequenced genomes (Supplementary Fig. S5). In addition, the *P*_OTU_ also differed significantly between environments. The *P*_OTU (100%)_ value based on the total host-associated samples was 4.6% whereas the *P*_OTU (100%)_ value for all the free-living samples was only 2.1%. The *P*_OTU (100%)_ values for the animal corpus and plant corpus environments were 28.3% and 23.7%, respectively, whereas the *P*_OTU (100%)_ values for sediment (non-saline), soil (non-saline), and water (non-saline) environments were only 2.3%, 2.9%, and 2.9%, respectively. *P*_OTU (98.6%)_ and *P*_OTU (97%)_ also showed similar patterns (Fig. 3b). Thus, despite the rapid accumulation of prokaryotic genomic information, the genome-sequenced proportion of the global prokaryotic taxa was still fairly low.

### The majority of the biomes were occupied by a few predominant taxa with high relative abundances

**Fig. 4:**
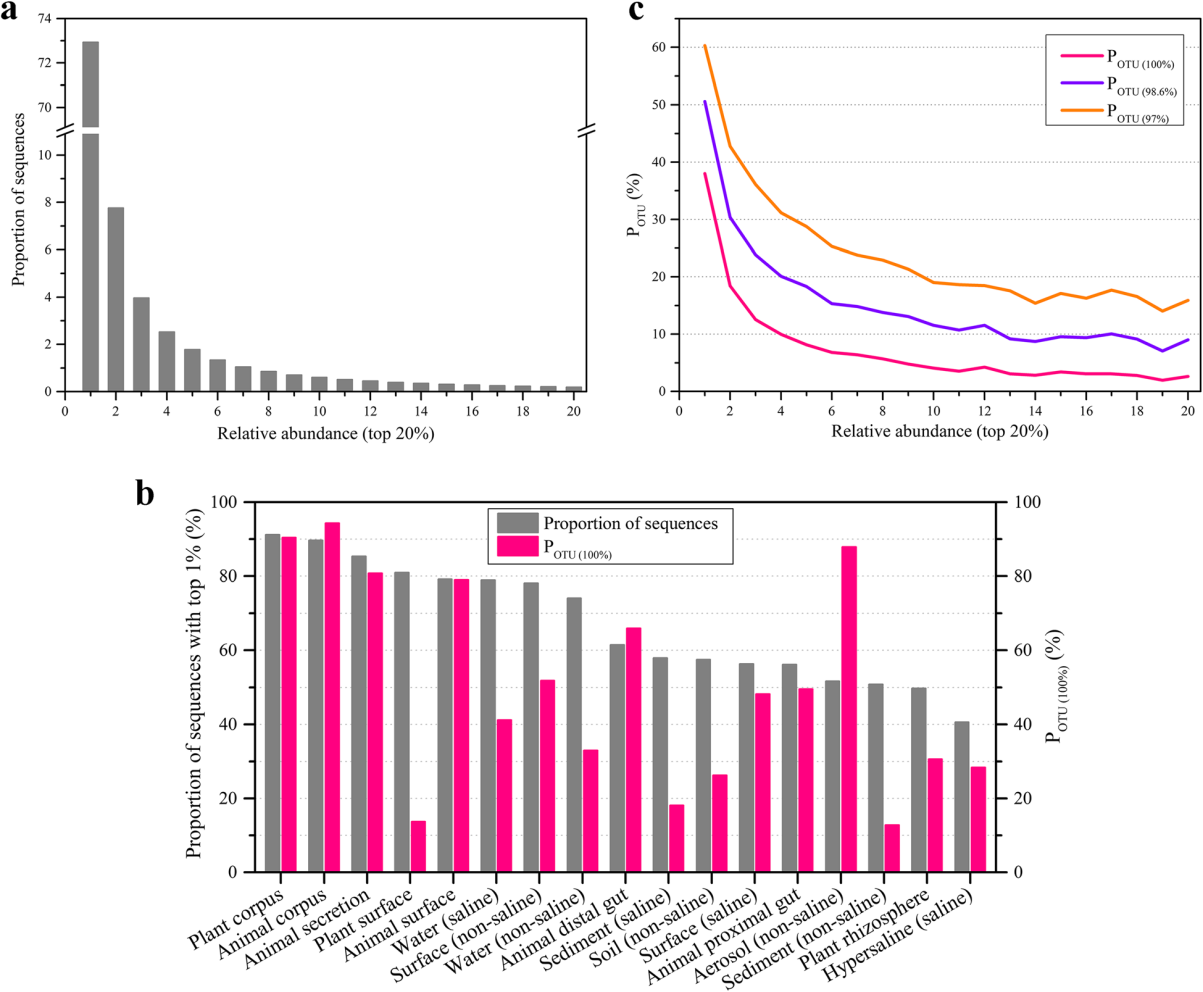
High genome-sequenced proportion of prokaryotic taxa with high abundance. **a** The top 1% of the prokaryotic taxa account for 72.9% of the global prokaryotic biomes. **b** The top 1% of the prokaryotic taxa from different environment types accounted for more than 40% with a genome-sequenced proportion greater than 10%. The gray column represents the cellular proportion of the top 1% of the taxa, and the red column represents the *P*_OTU (100%)_. **c** High genome-sequenced proportion of the top 1%. The red line is *P*_OTU (100%)_, the blue line is *P*_OTU (98.6%)_, and the orange line is *P*_OTU (97%)_

Our results showed that the top 1% of the prokaryotic taxa (sorted by their percentage of 16S rRNA sequences) accounted for 72.9% of the global prokaryotic biomes (Fig. 4a and Supplementary Fig. S6). These top 1% of taxa always had a high abundance in different environment types (Fig. 4b), which was similar to a recent report on global soil dominant bacteria [19]. By contrast, the rare taxa with low abundance (the total number of sequences < 10) accounted for 59.8% of the total prokaryotic taxa but only 1.2% of the global prokaryotic cells (Supplementary Fig. S7). We found that the number of samples affected the observed proportion of rare taxa to global taxa; as the number of samples increased, the ratio value increased gradually (Supplementary Fig. S8). Notably, the genome-sequenced proportion of the top 1% of prokaryotic taxa reached 38.0% whereas that of the 59.8% of prokaryotic taxa with a low abundance was only 0.6% (Fig. 4c and Supplementary Fig. S6). The genome-sequenced proportions of the top 1% of prokaryotic taxa from different environment types exceeded 12% (Fig. 4b). We further selected 1325 highly abundant and widely distributed OTUs on the following conditions: existing in at least 9 environment types and at least 100 samples and had an abundance reaching the top 1% in at least 1 type of environment (Supplementary dataset 3). These predominant taxa accounted for only 0.5% of the total OTUs but contributed to 50.3% of the global prokaryotic biomes. The genome-sequenced proportion was fairly high in these dominant taxa, and the *P*_OTU (100%)_, *P*_OTU (98.6%)_, and *P*_OTU (97%)_ values were 48.2%, 61.7%, and 71.3%, respectively (Supplementary Fig. S9). The majority of biomes were occupied by a few predominant taxa with high genome-sequenced proportions.

### Culturability altered genome-sequenced preferences among prokaryotes but not environments

We estimated the *P*_OTU_ values of prokaryotes at different taxonomic levels (Supplementary dataset 4), which showed that the *P*_OTU_ values were obviously different among different taxa, and the *P*_OTU_ value of the same taxon also differed significantly among different environment types (Supplementary Figs. S10, S11, S12, S13, S14 and S15). For example, of the 11 phyla with OTU numbers greater than 1%, the highest *P*_OTU (100%)_ value was 5.7% for *Actinobacteria*, and the lowest *P*_OTU (100%)_ value was 0.04% for *Parcubacteria*; the difference between them spanned more than 100-fold (Supplementary Fig. S10).

Due to improvements in sequencing throughput and computational techniques, cultivation-independent recovery of genomes from metagenomic data has rapidly developed. In total, 7903 bacterial and archaeal metagenome-assembled genomes (MAGs) were recovered from massive metagenomic data, which were considered from uncultivated strains [8]. We assessed the effect of strain culturability on the current genomic sequencing preferences using these MAGs and 155,810 cultured genomes (Supplementary dataset 5). The results showed that the genome-sequenced proportion of prokaryotes increased by 0.1% after combining these MAGs. According to the environment types, the *P*_OTU (100%)_ based on MAGs was highly positively correlated with that based on RefSeq (*r* = 0.91, *p* < 0.01) (Supplementary Fig. S16). The result showed that, similar to the RefSeq genomes, the MAGs also showed environmental differences, and the culturability of strains was not the main factor leading to these differences. For the 11 phyla with an OTU number proportion greater than 1%, there was no significant correlation between the *P*_OTU (100%)_ based on the MAGs and the RefSeq (*p* > 0.05) (Supplementary Fig. S16). This indicated that although the recovered MAGs had a distinct difference in prokaryotic taxa, its species preference was significantly different from the RefSeq genomes.

## Discussion

The genome is the basic resource for understanding the physiology, ecology, and evolution of prokaryotes. More than 200,000 bacterial and archaeal genomes are now available from over two decades of development [3, 6]. These genomes provide important insights into the role of microorganisms in industrial processes, the pathogenic mechanisms of pathogenic microorganisms, etc. In this study, we assessed the genome-sequenced proportion of global prokaryotes. We found that the median proportions of the genome-sequenced prokaryotic cells and taxa (at 100% identities in the 16S-V4 region) in global biomes were 38.1% (16.4–86.3%) and 18.8% (9.1–52.6%), respectively. The *B*_cell (97%)_ of 61.9% of the samples reached 50%, and the *B*_OTU (97%)_ of 38.4% of the samples reached 50% after combining closely related strains. In addition, the median *B*_cell (97%)_ and *B*_OTU (100%)_ values in host-associated biomes were 85.6% (43.2–98.0%) and 62.8% (9.8–82.3%), respectively, which were significantly higher than those in free-living biomes. Thus, the genetic information of a specific prokaryotic biome may have been reported to a considerable degree.

However, compared to prokaryotic biomes, the genome-sequenced proportion of global prokaryotic OTUs was fairly low. Our results suggest that only 2.1% of the global prokaryotic taxa (at 100% identities in the 16S-V4 region) have been sequenced. More than 75% of prokaryotic OTUs could exist in multiple biomes; the more types of environments in which prokaryotic OTUs can survive, the higher the genome-sequenced proportion could be. Prokaryotic biomes are usually composed of a few predominant taxa with a high abundance and many rare taxa with a low abundance [20, 21]. We found that 0.5% of predominant OTUs occupied 50.3% of prokaryotic cell abundance with a high genome-sequenced proportion (48.2%); however, the 60% of rare OTUs only accounted for 1.2% of the global prokaryotic cells with a low genome-sequenced proportion (0.6%). A large number of rare taxa are considered to be critical components of the earth’s ecosystem and contain a large functional genes pool [21, 22]. Therefore, from this perspective, our current understanding of global prokaryotic genomic information remains very limited due to the large number of genome-unsequenced rare taxa, and the exploration of this huge genetic resource is just beginning.

Predominant taxa are considered the priority for isolated culture and genome sequencing [19]. We identified 1325 predominant OTUs with a wide distribution, high abundance, and adaptability to a variety of environmental types, more than half of which had not been genome-sequenced. In particular, some predominant taxa acquired less attention in specific environmental types. For example, the top 1% taxa of abundance in plant surfaces and animal surfaces accounted for 81.0% and 79.2% of the global prokaryotic biomes whereas the genome-sequenced proportions of the taxa were only 13.7% and 79.0%, respectively. The *P*_OTU (100%)_ of plant surfaces (leaf or kelp surface biofilms) was ranked 8th, but its median *B*_cell_ was last given the lack of understanding of predominant taxa.

Currently, most of the prokaryotic sequenced genomes (RefSeq genomes) are from pure cultures, while MAGs are not limited by culturability [8, 9, 23]. We found similar genome-sequenced differences among different environment types between RefSeq genomes and MAGs, which indicated that the current imbalance of prokaryotic genome sequencing in different environments was more likely due to differences in researchers’ attention rather than prokaryotic culturability. Although the significant genome-sequenced differences among different taxa between RefSeq genomes and MAGs suggested that culturability caused genomic sequencing preferences had no effect on MAGs, MAGs had also owned its own taxa sequenced preferences.

The paradigm that only 1% of prokaryotes are culturable has a profound impact on microbial ecology but has recently been debated [24–26]. Since the RefSeq genomes are mainly from culturable taxa, and a significant proportion of culturable taxa have not been sequenced, we estimate that the culturable rate of global prokaryotic taxa (> 97% identities) would be higher than the genome-sequenced proportion of 12.2%. Similar to the higher genome-sequenced proportion of the high abundance predominant taxa, predominant taxa should also have a much higher culturability rate than rare taxa; thus, the culturability rate of prokaryotic cells will be much higher than that of taxa. Consequently, our data indicated that the paradigm that only 1% of prokaryotes are culturable is out of date, both for cells and taxa.

## Conclusions

This study performed an in-depth analysis of the prokaryotic genome-sequenced proportion in the EMP and comprehensively showed the global-scale genome-sequenced degree for various environment types and different species. Most of the biomes were occupied by a few widespread predominant taxa. Given the high genome-sequenced proportion of predominant taxa, the genetic information of most prokaryotic biomes has been revealed to a high degree. However, due to the large number of rare taxa with unknown genomes, our current understanding of the global prokaryotic genome information remains limited. These results will be helpful for more reasonable and efficient explorations of prokaryotic genomes and will accelerate the comprehensive understanding of microbial ecological functions in different environments.

## Methods

### Data collection from EMP and RefSeq

The Earth Microbiome Project (EMP) was founded in 2010 to sample the Earth’s microbial communities at an unprecedented scale to advance our understanding of the organizing biogeographic principles that govern microbial community structure on Earth [13–15]. A total of 262,011 OTUs and their abundance and nucleic acid sequence information were collected from the website (ftp://ftp.microbio.me/emp/release1), which were obtained and shared by the EMP from 10,000 samples using the Deblur software [27]. Chimera filtering relied on the EMP project. The NCBI’s reference sequence (RefSeq) database is a curated non-redundant collection of sequences representing whole or frame genomes [28]. We obtained all of the 155,810 bacterial or archaeal genomes collected by the database before July 2019. In addition, 7903 (1539 contained the 16S rRNA gene) metagenome-assembled genomes (MAGs) [8] recovered from > 1500 public metagenomes using MetaBAT [29] were also collected for representative uncultivated bacteria and archaea.

### Sequence alignment and analysis

Alignment between the EMP OTUs and 155,810 or 7903 genomes was performed using BLASTn (*E* value < 1e–5) [30]. To assess the adequacy of the OTUs, we analyzed all the samples by increasing the number of samples from 1000 to 10,000 randomly. The genome-sequenced proportions of cells and taxa (at 100%, > 98.6% or, > 97% identities in the 16S-V4 region) in a specific prokaryotic biome were defined as *B*_cell_ and *B*_OTU_, respectively. The genome-sequenced proportion of taxa (at 100%, > 98.6%, or > 97% identities in the 16S-V4 region) from subgroup or global biomes was defined as the *P*_OTU_. The 100% identity represents the most rigorous and accurate match, while 98.6% and 97% identities are the new and traditional criteria for species definitions, respectively [16–18]. Briefly, *B*_cell_ represents the ratio of the genome-sequenced sequences in a single sample, *B*_OTU_ represents the ratio of the genome-sequenced OTUs in a single sample and *P*_OTU_ represents the ratio of the genome-sequenced OTUs in multiple samples.

### Taxonomic analysis of EMP OTU

The taxonomy of each OTU was analyzed by the Ribosomal Database Project (RDP) Classifier [31] at a 70% confidence threshold. The EMP ontology (EMPO) classified 17 microbial environments (level 3) as free living or host associated (level 1) and saline or non-saline (if free living) or animal or plant (if host associated) (level 2) [13]. Based on the taxonomic results and the EMPO (level 3) for each OTU, we calculated the composition and relative abundance of different levels of taxonomy (phylum, class, order, family, and genus) in different environments.

## Supplementary information

**Additional file 1 : Supplementary Fig. S1.** Genome-sequenced degree of prokaryotic biomes. **a**, Genome-sequenced proportion of cells. **b**, Genome-sequenced proportion of taxa. OTUs share at least 98.6% identities with the sequenced genomes. Based on the analysis of 10,000 EMP samples, each grey point represents a single sample. For the box plots, the middle line indicates the median, the box represents the 25th–75th percentiles, and the error bars represent the 10th–90th percentiles of observations. Environment types were classified by EMPO; red represents host-associated and green represents free-living.

**Additional file 2: Supplementary Fig. S2.** Genome-sequenced degree of prokaryotic biomes. **a**, Genome-sequenced proportion of cells. **b**, Genome-sequenced proportion of taxa. OTUs share at least 97% identities with the sequenced genomes. Based on the analysis of 10,000 EMP samples, each grey point represents a single sample. For the box plots, the middle line indicates the median, the box represents the 25th–75th percentiles, and the error bars indicate the 10th–90th percentiles of observations. Environment types were classified by EMPO; red represents host-associated and green represents free-living.

**Additional file 3: Supplementary Fig. S3.** Genome-sequenced proportions of prokaryotic biomes are significantly negatively correlated with the biomes’ alpha diversity indices. Alpha diversity indices include observed OTUs, Shannon index, Chao1 index, and Faith's PD value. Each point represents one single sample from a total of 10,000 samples. Brown represents B_cell (100%)_ and purple represents B_OTU (100%)_.

**Additional file 4: Supplementary Fig. S4.** Genome-sequenced proportion of global prokaryotic taxa. As the number of samples increases, the P_OTU (98.6%)_ and P_OTU (97%)_ values show an exponential declining trend and finally stabilize at 6.8% and 12.2%, respectively. A random selection of 1000, 2000…, 9000 samples was performed 10 times for each group to calculate the mean value and standard deviation. Blue represents P_OTU (98.6%)_ and orange represents P_OTU (97%)_.

**Additional file 5: Supplementary Fig. S5.** The trends of P_OTU_ over time and genome number. **a**, The P_OTU_ has grown exponentially over time. **b**, The P_OTU_ shows an allometric rising trend as the number of sequenced genomes increases. Red represents P_OTU (100%)_, blue represents P_OTU (98.6%)_ and orange represents P_OTU (97%)_.

**Additional file 6: Supplementary Fig. S6.** The top 1% of the prokaryotic taxa accounts for 72.9% of the global prokaryotic biomes with high genome-sequenced proportion. The red line is P_OTU (100%)_, the blue line is P_OTU (98.6%)_, and the orange line is P_OTU (97%)_.

**Additional file 7: Supplementary Fig. S7.** The rare taxa with low abundance (total number of sequences < 10) account for 59.8% of the total prokaryotic taxa but only 1.2% of the global prokaryotic cells with a 0.6% genome-sequenced proportion.

**Additional file 8: Supplementary Fig. S8.** The proportion of rare taxa to the global taxa increases gradually and stabilizes at approximately 60% as the number of samples increases.

**Additional file 9: Supplementary Fig. S9.** Predominant taxa have a high abundance and wide distribution. **a**, A total of 1,325 OTUs were selected according to the following conditions: existing in at least 9 environments and at least 100 samples and an abundance reaching the top 1% in at least 1 environmental type. **b**, OTU number proportion and P_OTU (100%)_ (in brackets) of the main phylum in the predominant taxa. **c**, OTU number proportion and P_OTU (100%)_ (in brackets) of the main class in the predominant taxa. **d**, OTU number proportion and P_OTU (100%)_ (in brackets) of the main order in the predominant taxa.

**Additional file 10: Supplementary Fig. S10.** Obviously genome-sequenced preferences of prokaryotes among taxa. **a**, The OTU number proportion of the 11 main phyla. **b**, The P_OTU (100%)_ of the 11 main phyla.

**Additional file 11: Supplementary Fig. S11.** Heatmap of P_OTU (100%)_ between the 11 main phyla and the 17 environment types.

**Additional file 12: Supplementary Fig. S12.** Genome-sequenced proportion of 46 main classes of prokaryotic predominant taxa. The parenthesis shows the OTU number proportion of each class in all prokaryotic OTUs.

**Additional file 13: Supplementary Fig. S13.** Genome-sequenced proportion of the 49 main orders of prokaryotic predominant taxa. The parenthesis shows the OTU number proportion of each order in all prokaryotic OTUs.

**Additional file 14: Supplementary Fig. S14.** Genome-sequenced proportion of the 58 main families of prokaryotic predominant taxa. The parenthesis shows the OTU number proportion of each family in all prokaryotic OTUs.

**Additional file 15: Supplementary Fig. S15.** Genome-sequenced proportion of 55 main genera of prokaryotic predominant taxa. The parenthesis shows the OTU number proportion of each genus in all prokaryotic OTUs.

**Additional file 16: Supplementary Fig. S16.** Culturability alters genome-sequenced preferences among prokaryotes but not environments. **a**, For the 17 environment types, the P_OTU (100%)_ based on MAGs is highly positively correlated with that based on RefSeq (*r* = 0.91, *p* < 0.01). **b**, For the 11 phyla with an OTU number proportion greater than 1%, the P_OTU (100%)_ based on the MAGs has no significant correlation with that based on the RefSeq (*p* > 0.05).

**Additional file 17: Supplementary dataset 1.** B_cell_, B_OTU_, alpha diversity index and environment type of 10,000 samples.

**Additional file 18: Supplementary dataset 2.** B_cell_, B_OTU_ and P_OTU_ of different environment types.

**Additional file 19: Supplementary dataset 3.** Summary of 262,011 OTUs and predominant taxa.

**Additional file 20: Supplementary dataset 4.** The P_OTU_ of different taxonomic levels (domain, phylum, class, order, family and genus) in different environment types.

**Additional file 21: Supplementary dataset 5.** The P_OTU_ of prokaryote-based MAGs.

## Data Availability

The main data supporting the findings of this study are available within the article and in its Supplementary Information. All other data supporting the findings of this study are available from the corresponding authors upon reasonable request.
