# Supplementary figures and images for: Estimate of the sequenced proportion of the global prokaryotic genome
Source: Microbiome. 2020 Sep 16;8:134. doi: 10.1186/s40168-020-00903-z (PMC7496214; doi:10.1186/s40168-020-00903-z)

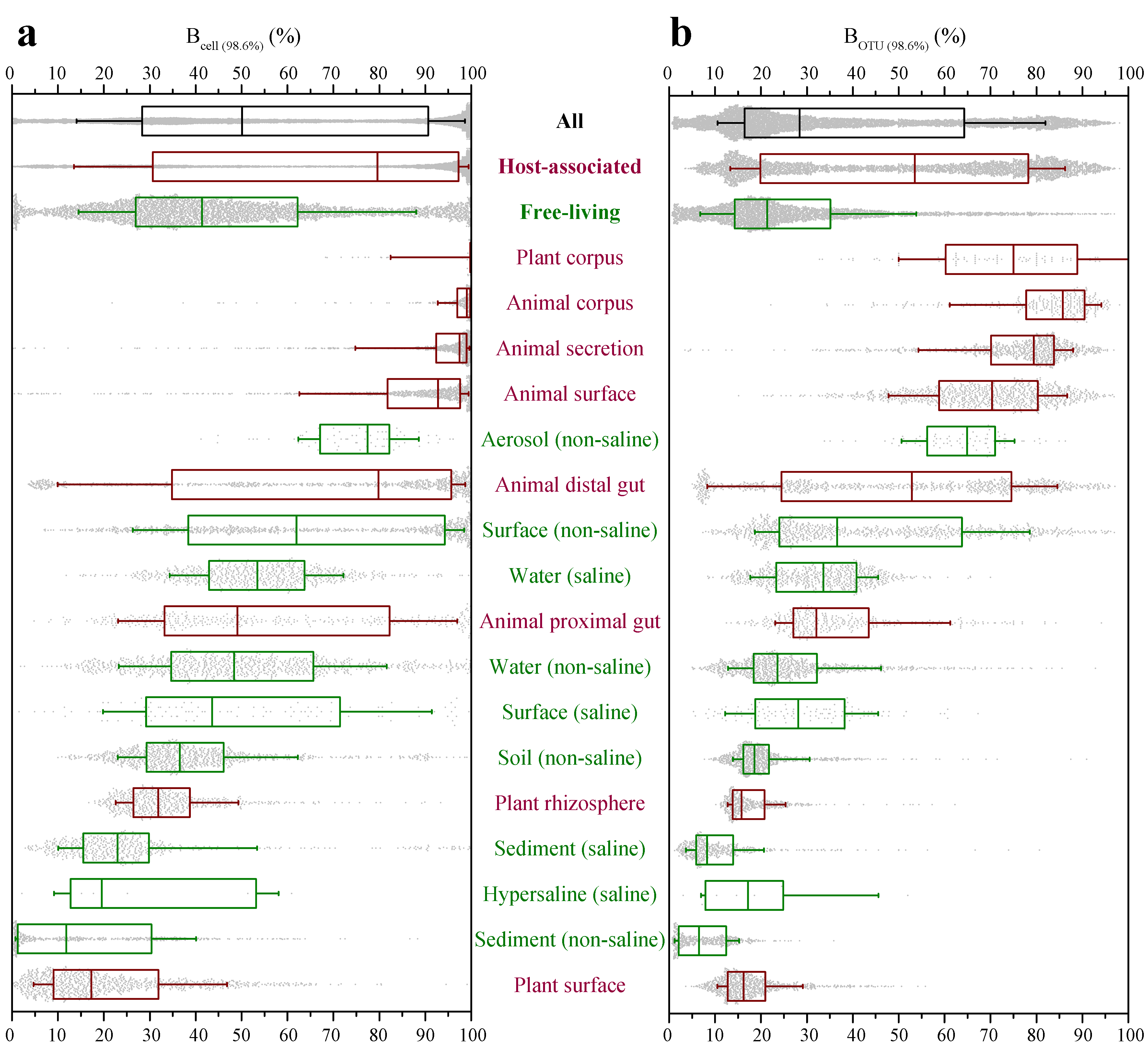

Supplement: Supplementary file 2 — Additional file 1 : Supplementary Fig. S1. Genome-sequenced degree of prokaryotic biomes. a, Genome-sequenced proportion of cells. b, Genome-sequenced proportion of taxa. OTUs share at least 98.6% identities with the sequenced genomes. Based on the analysis of 10,000 EMP samples, each grey point represents a single sample. For the box plots, the middle line indicates the median, the box represents the 25th–75th percentiles, and the error bars represent the 10th–90th percentiles of observations. Environment types were classified by EMPO; red represents host-associated and green represents free-living. [file 40168_2020_903_MOESM1_ESM.tif]

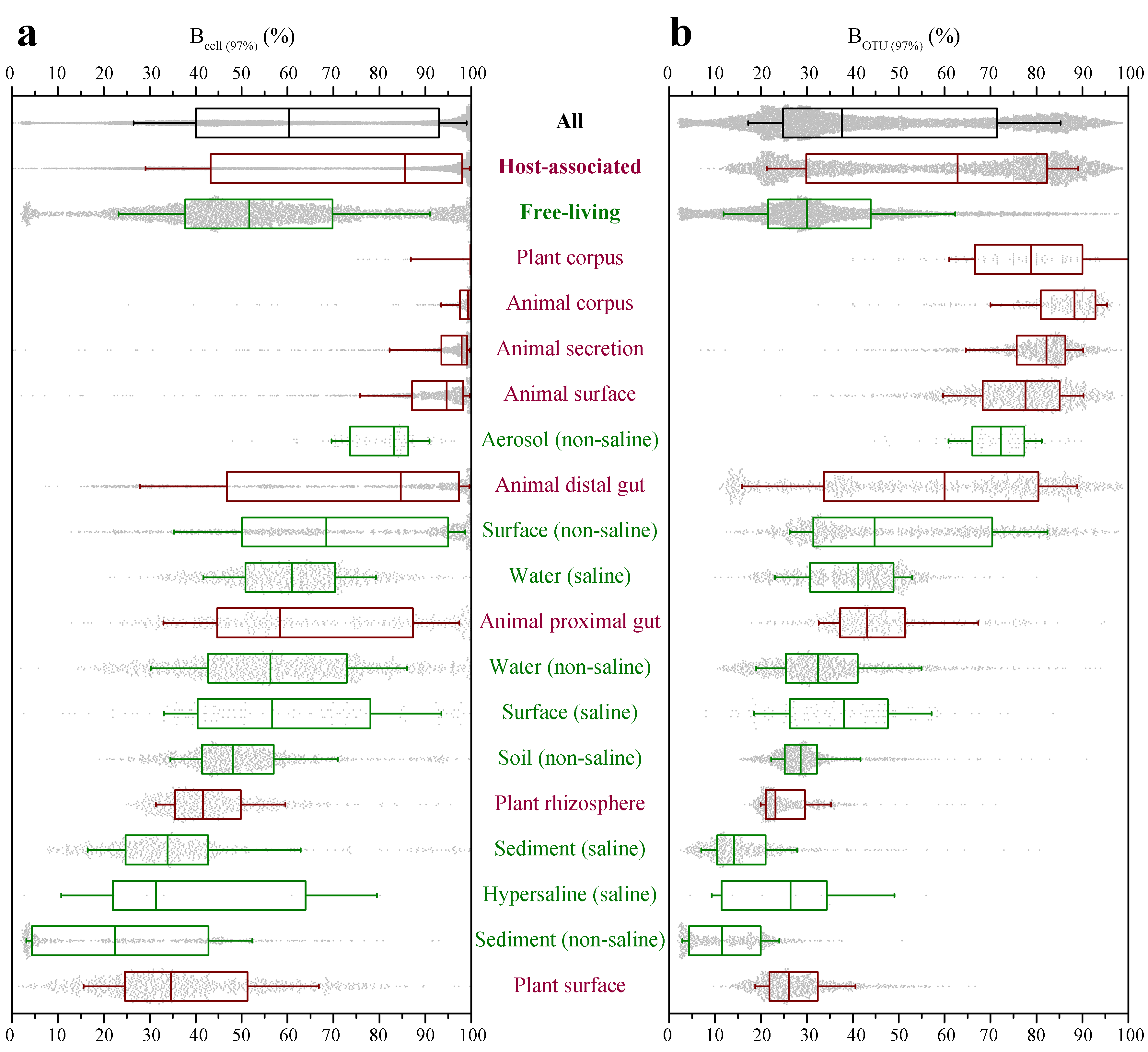

Supplement: Supplementary file 3 — Additional file 2: Supplementary Fig. S2. Genome-sequenced degree of prokaryotic biomes. a, Genome-sequenced proportion of cells. b, Genome-sequenced proportion of taxa. OTUs share at least 97% identities with the sequenced genomes. Based on the analysis of 10,000 EMP samples, each grey point represents a single sample. For the box plots, the middle line indicates the median, the box represents the 25th–75th percentiles, and the error bars indicate the 10th–90th percentiles of observations. Environment types were classified by EMPO; red represents host-associated and green represents free-living. [file 40168_2020_903_MOESM2_ESM.tif]

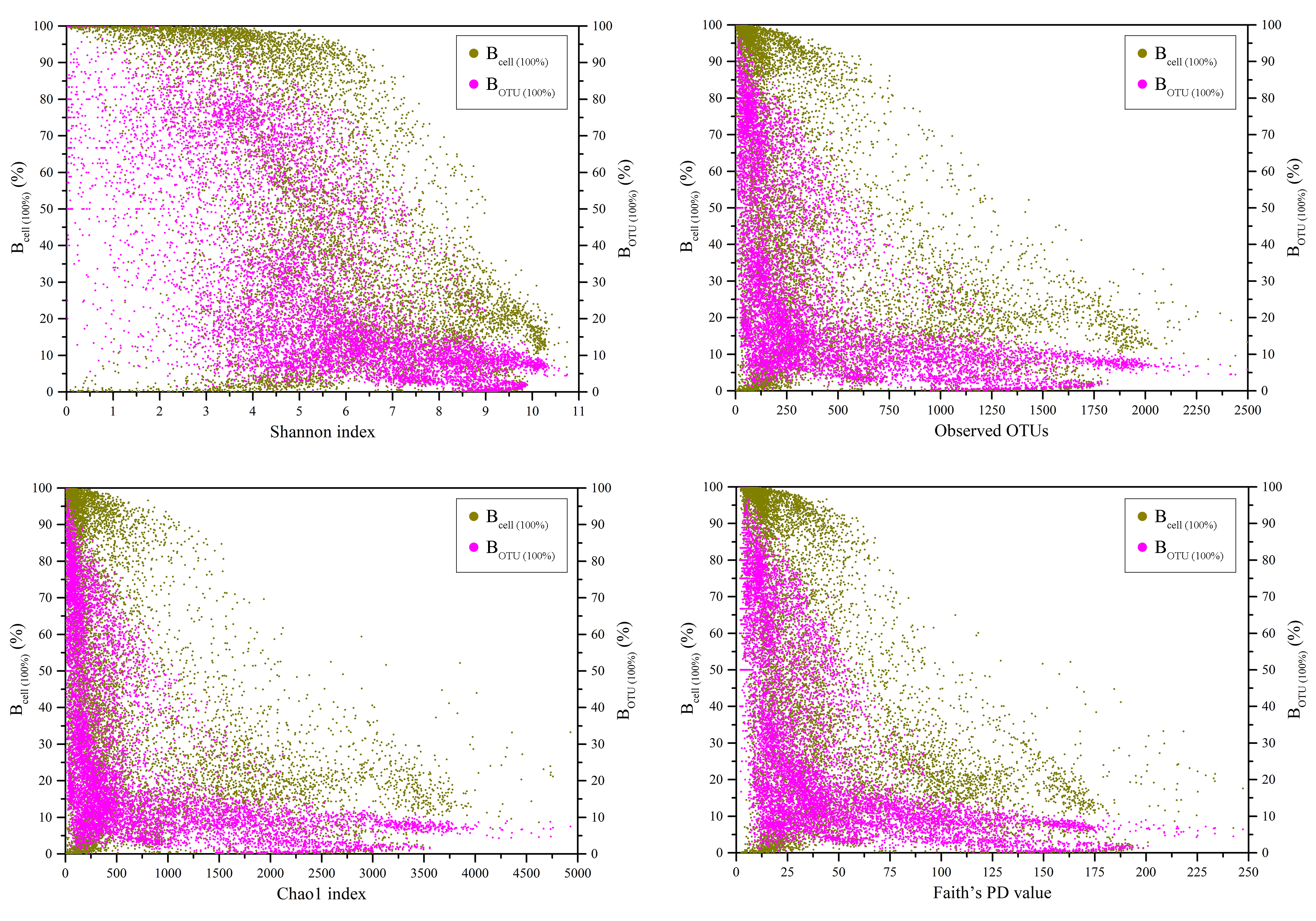

Supplement: Supplementary file 4 — Additional file 3: Supplementary Fig. S3. Genome-sequenced proportions of prokaryotic biomes are significantly negatively correlated with the biomes’ alpha diversity indices. Alpha diversity indices include observed OTUs, Shannon index, Chao1 index, and Faith's PD value. Each point represents one single sample from a total of 10,000 samples. Brown represents Bcell (100%) and purple represents BOTU (100%). [file 40168_2020_903_MOESM3_ESM.tif]

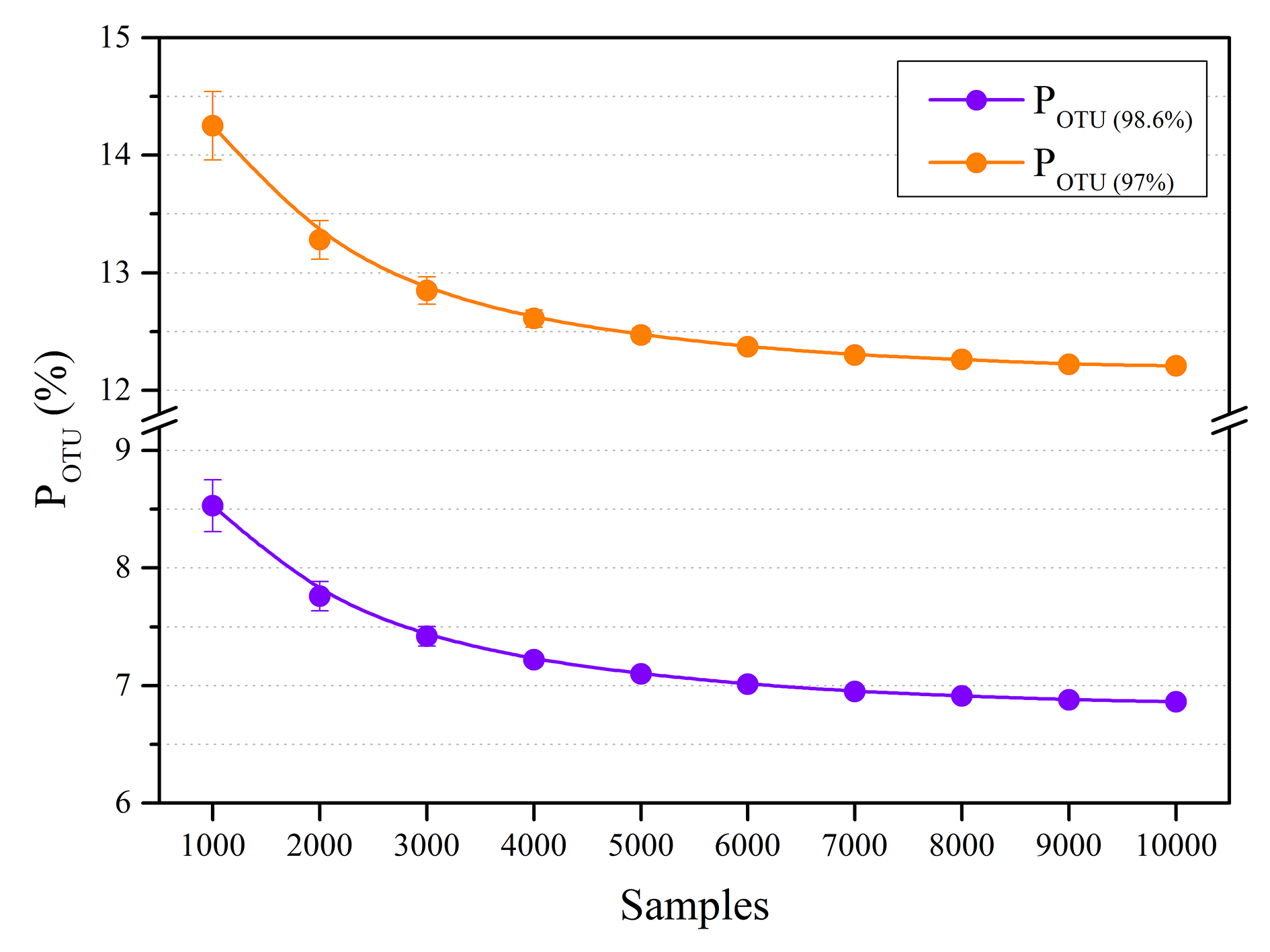

Supplement: Supplementary file 5 — Additional file 4: Supplementary Fig. S4. Genome-sequenced proportion of global prokaryotic taxa. As the number of samples increases, the POTU (98.6%) and POTU (97%) values show an exponential declining trend and finally stabilize at 6.8% and 12.2%, respectively. A random selection of 1000, 2000…, 9000 samples was performed 10 times for each group to calculate the mean value and standard deviation. Blue represents POTU (98.6%) and orange represents POTU (97%). [file 40168_2020_903_MOESM4_ESM.tif]

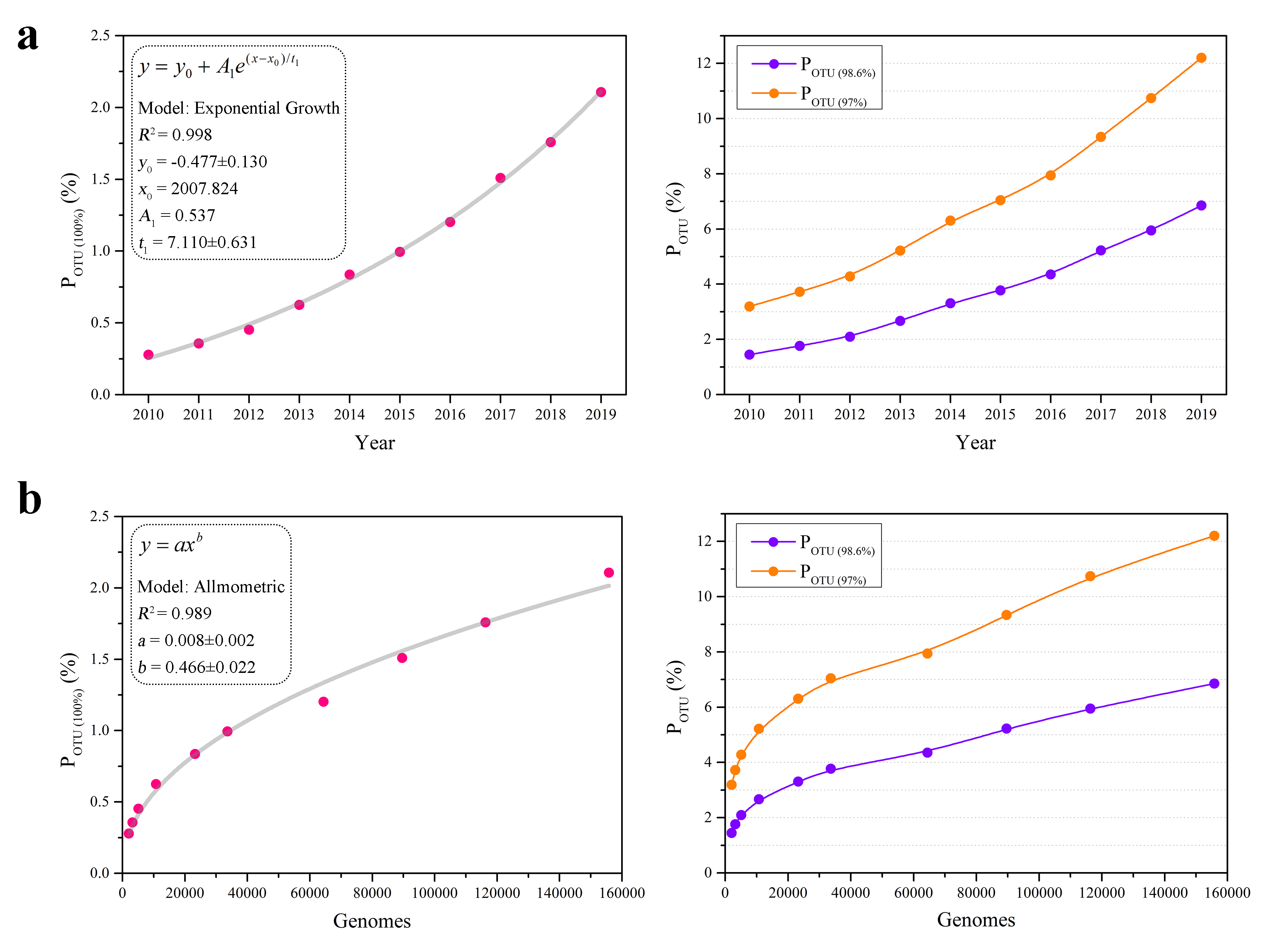

Supplement: Supplementary file 6 — Additional file 5: Supplementary Fig. S5. The trends of POTU over time and genome number. a, The POTU has grown exponentially over time. b, The POTU shows an allometric rising trend as the number of sequenced genomes increases. Red represents POTU (100%), blue represents POTU (98.6%) and orange represents POTU (97%). [file 40168_2020_903_MOESM5_ESM.tif]

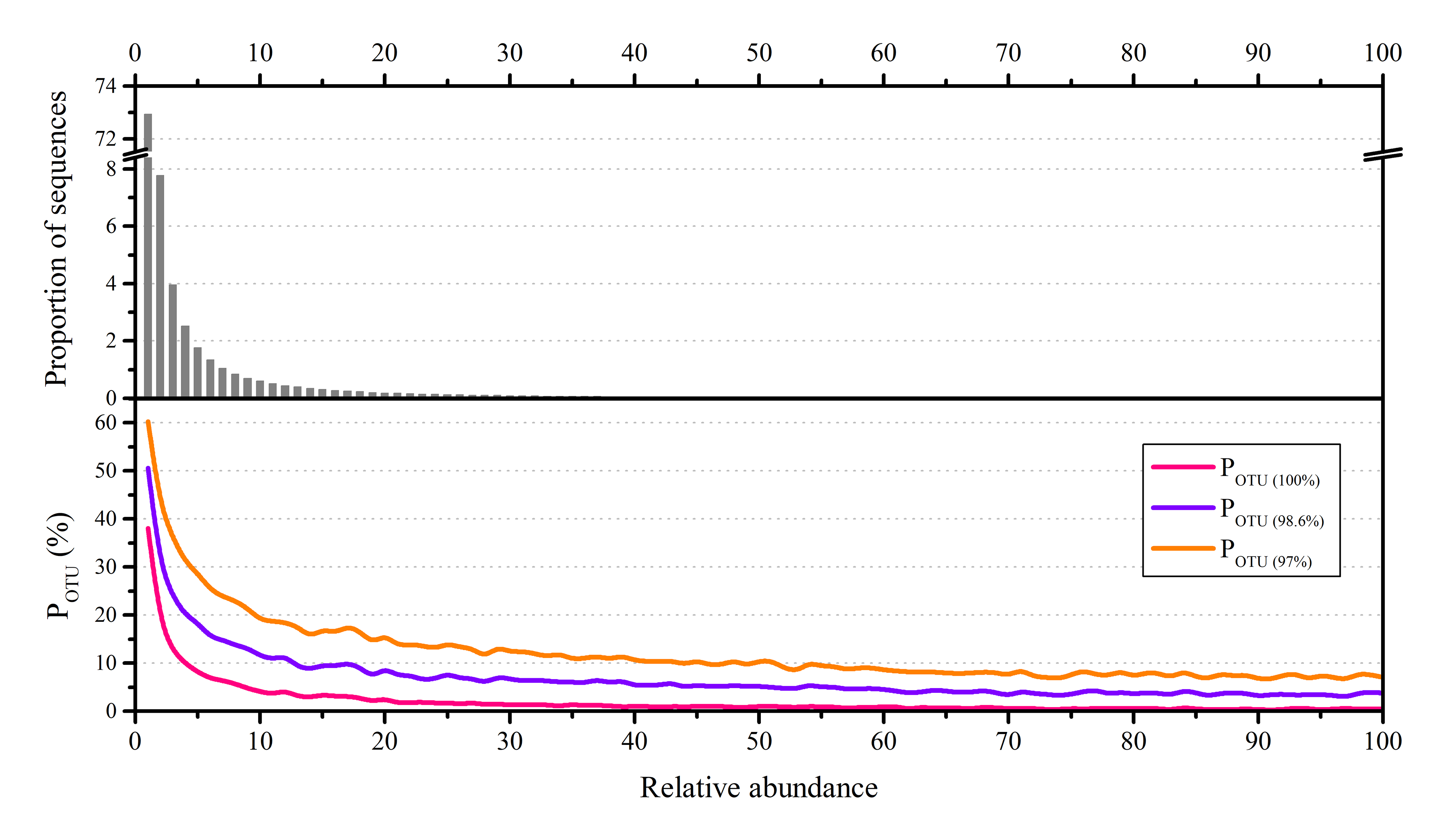

Supplement: Supplementary file 7 — Additional file 6: Supplementary Fig. S6. The top 1% of the prokaryotic taxa accounts for 72.9% of the global prokaryotic biomes with high genome-sequenced proportion. The red line is POTU (100%), the blue line is POTU (98.6%), and the orange line is POTU (97%). [file 40168_2020_903_MOESM6_ESM.tif]

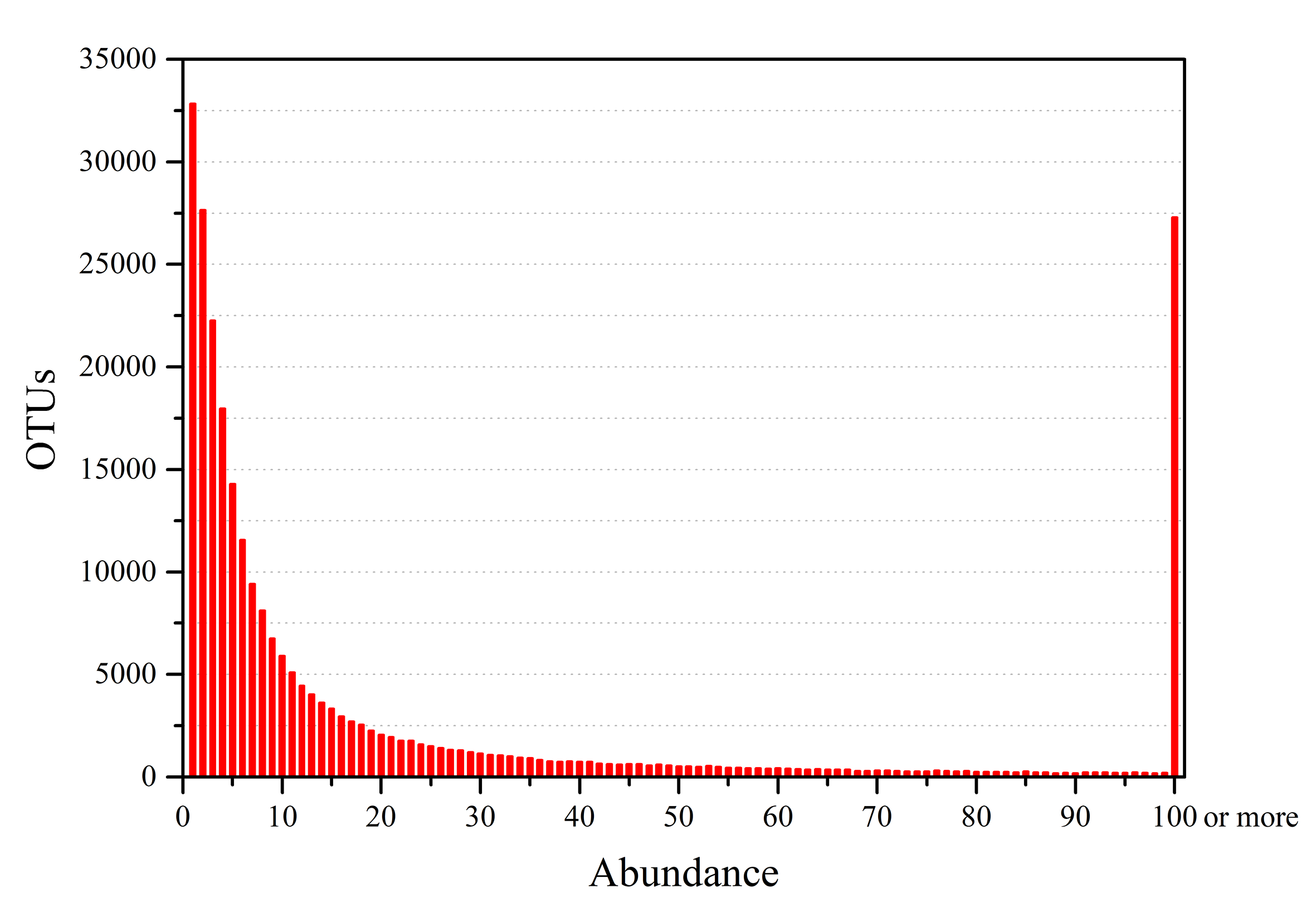

Supplement: Supplementary file 8 — Additional file 7: Supplementary Fig. S7. The rare taxa with low abundance (total number of sequences < 10) account for 59.8% of the total prokaryotic taxa but only 1.2% of the global prokaryotic cells with a 0.6% genome-sequenced proportion. [file 40168_2020_903_MOESM7_ESM.tif]

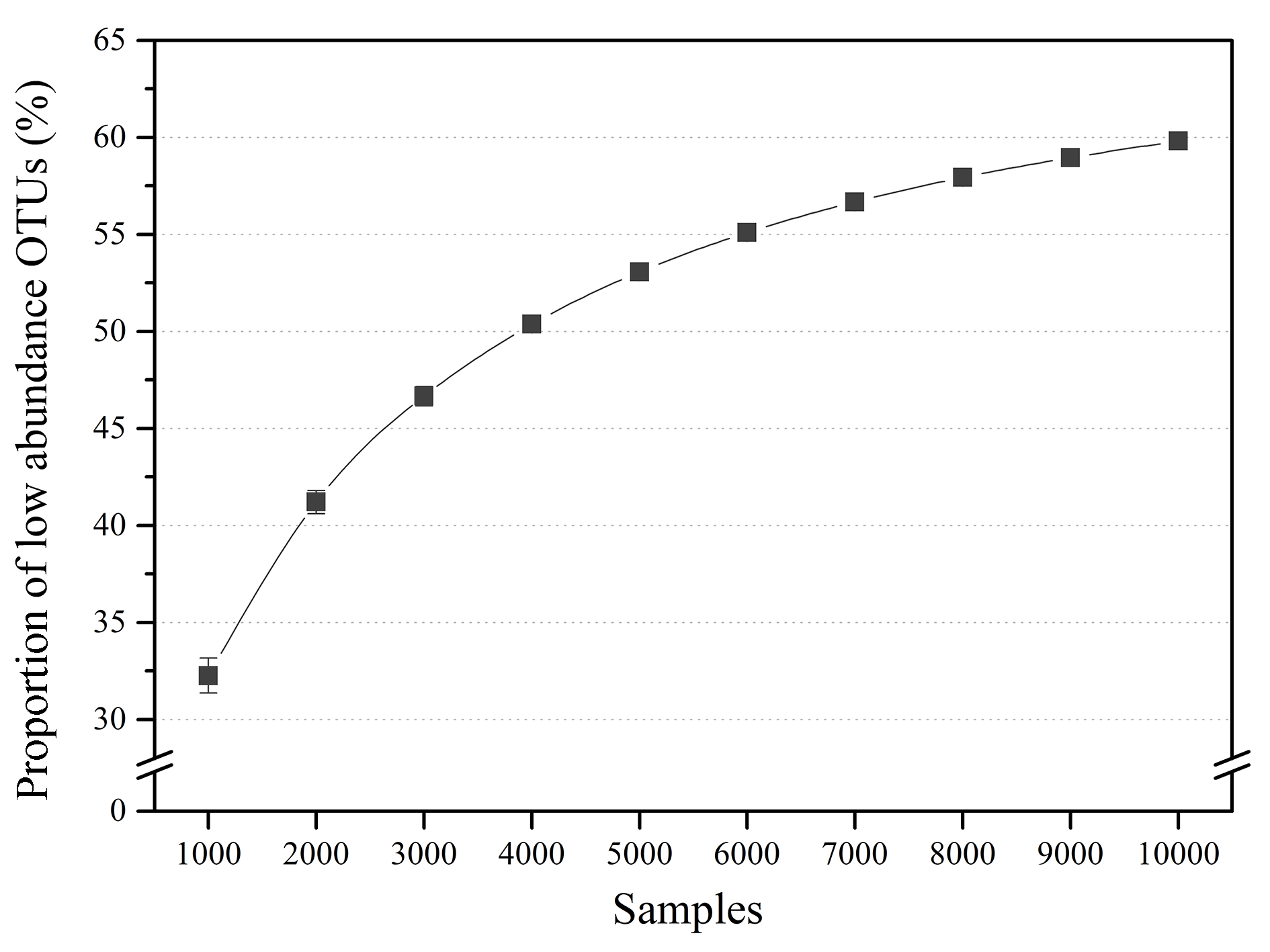

Supplement: Supplementary file 9 — Additional file 8: Supplementary Fig. S8. The proportion of rare taxa to the global taxa increases gradually and stabilizes at approximately 60% as the number of samples increases. [file 40168_2020_903_MOESM8_ESM.tif]

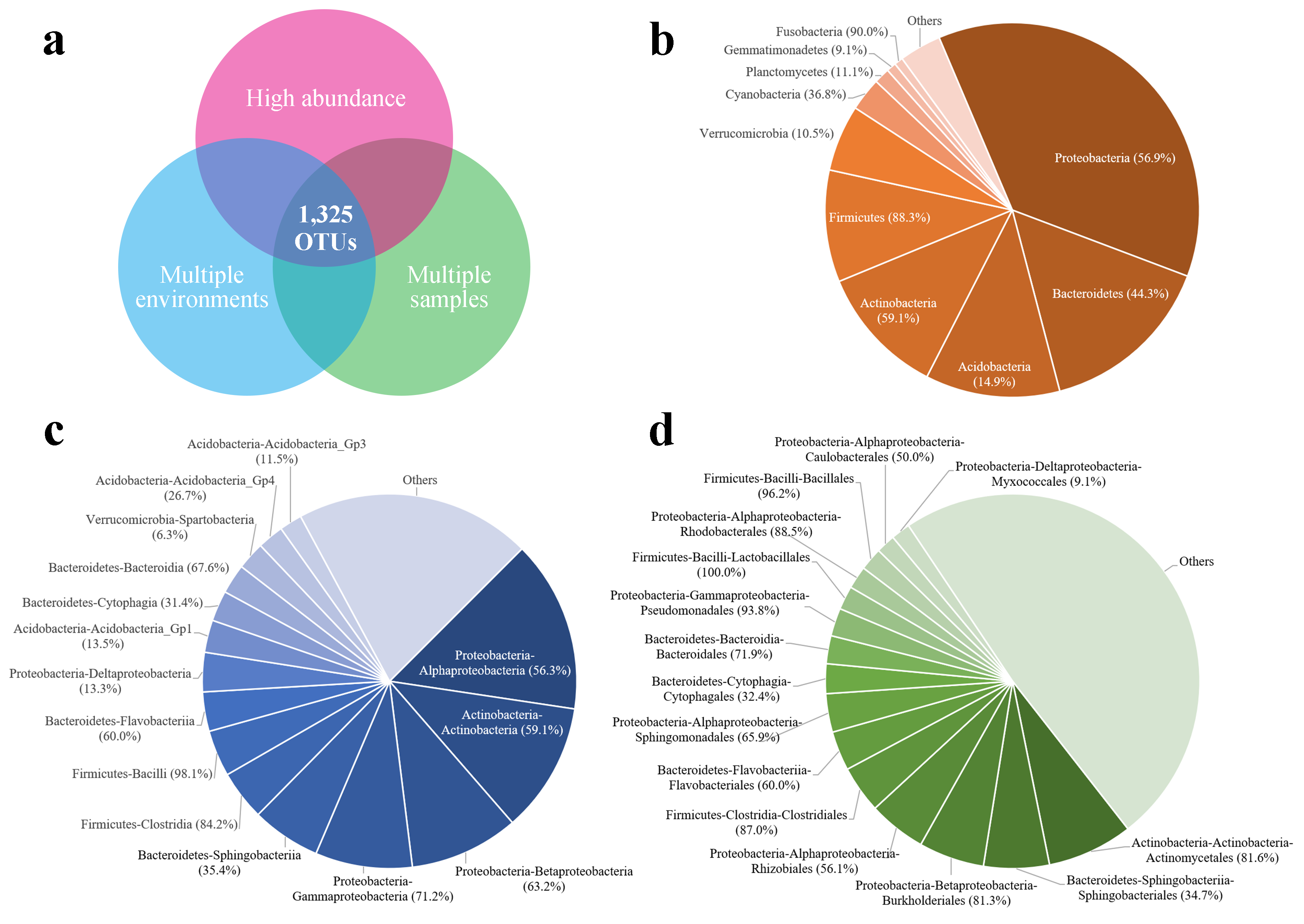

Supplement: Supplementary file 10 — Additional file 9: Supplementary Fig. S9. Predominant taxa have a high abundance and wide distribution. a, A total of 1,325 OTUs were selected according to the following conditions: existing in at least 9 environments and at least 100 samples and an abundance reaching the top 1% in at least 1 environmental type. b, OTU number proportion and POTU (100%) (in brackets) of the main phylum in the predominant taxa. c, OTU number proportion and POTU (100%) (in brackets) of the main class in the predominant taxa. d, OTU number proportion and POTU (100%) (in brackets) of the main order in the predominant taxa. [file 40168_2020_903_MOESM9_ESM.tif]

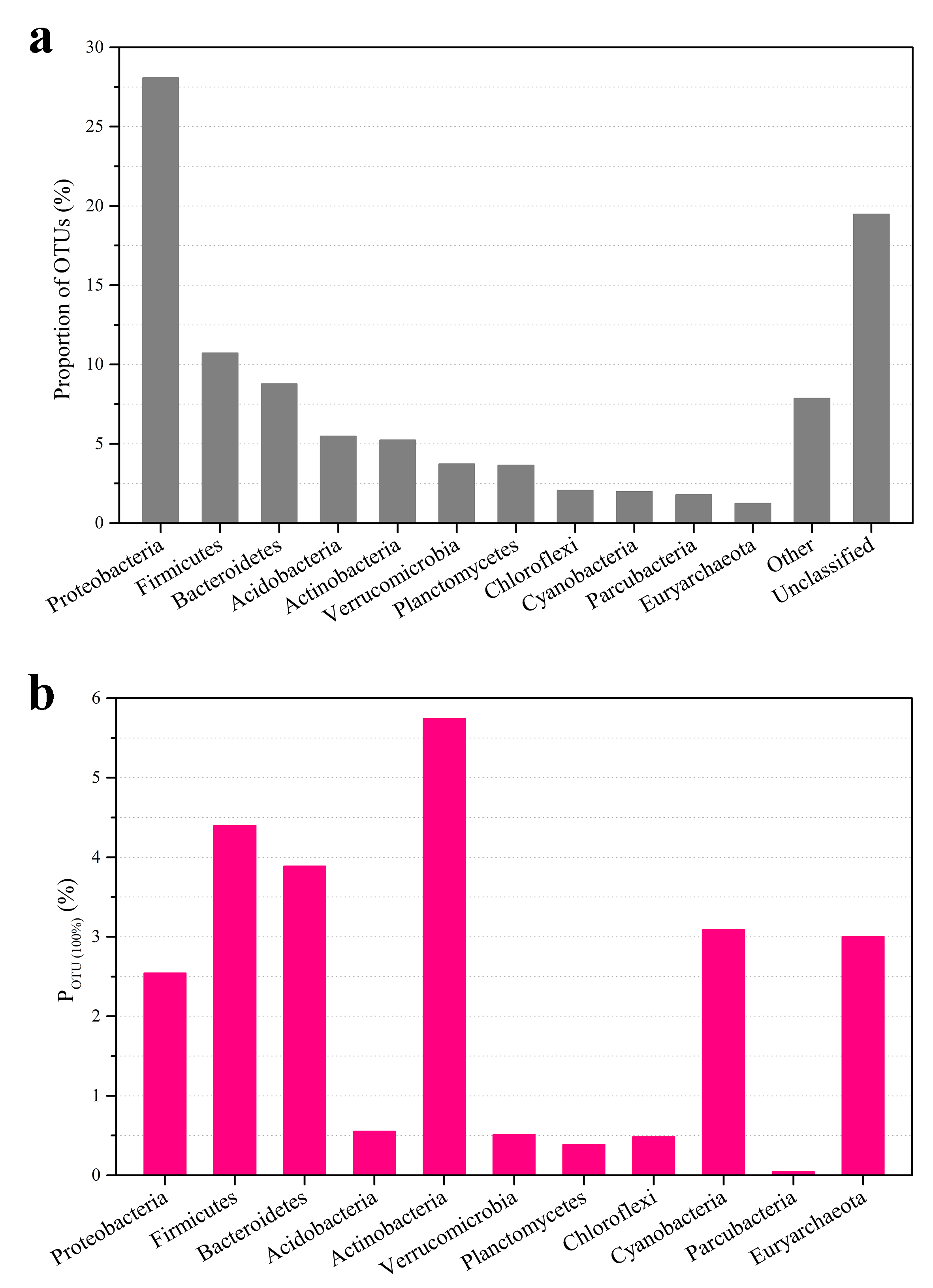

Supplement: Supplementary file 11 — Additional file 10: Supplementary Fig. S10. Obviously genome-sequenced preferences of prokaryotes among taxa. a, The OTU number proportion of the 11 main phyla. b, The POTU (100%) of the 11 main phyla. [file 40168_2020_903_MOESM10_ESM.tif]

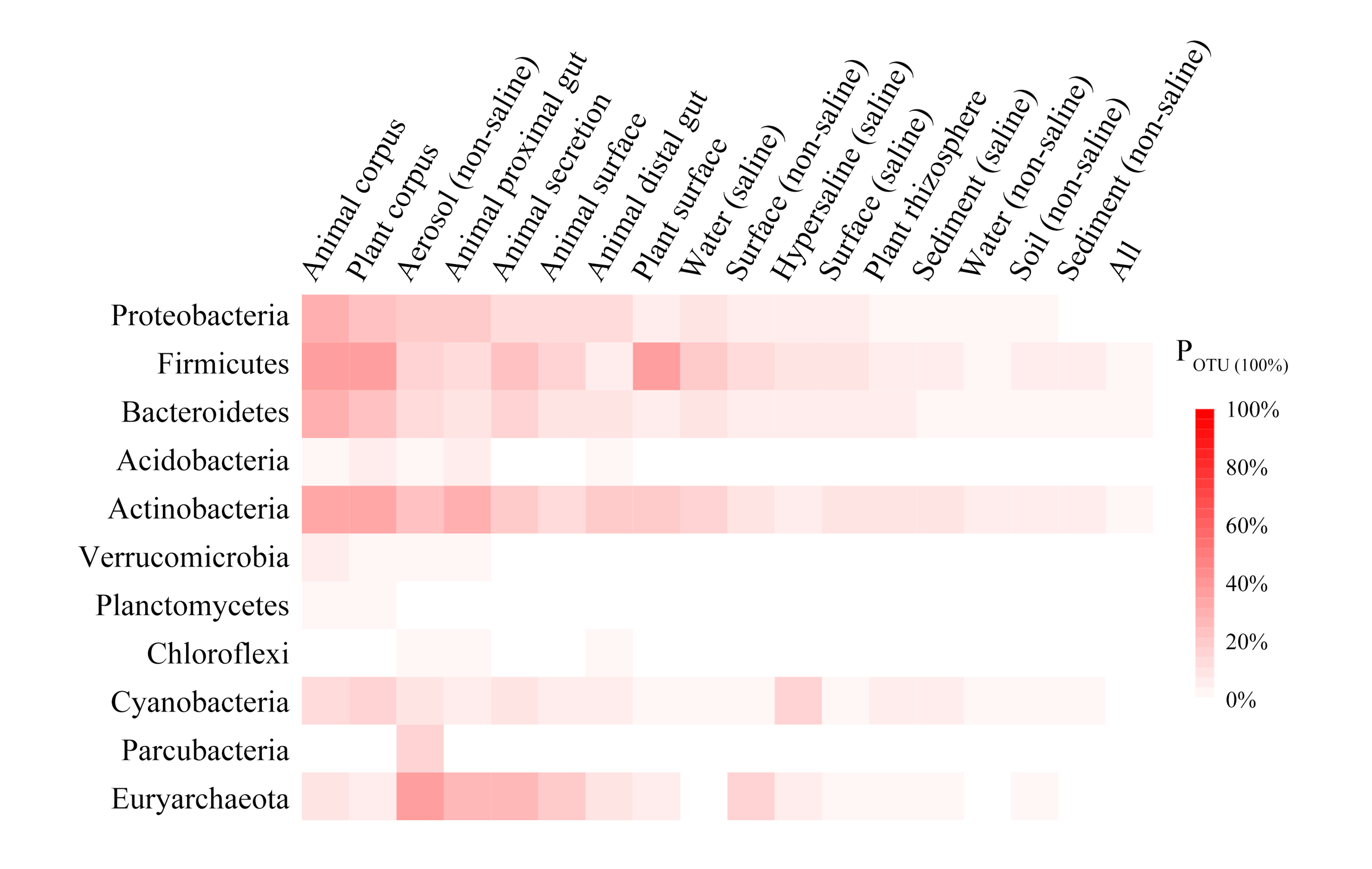

Supplement: Supplementary file 12 — Additional file 11: Supplementary Fig. S11. Heatmap of POTU (100%) between the 11 main phyla and the 17 environment types. [file 40168_2020_903_MOESM11_ESM.tif]

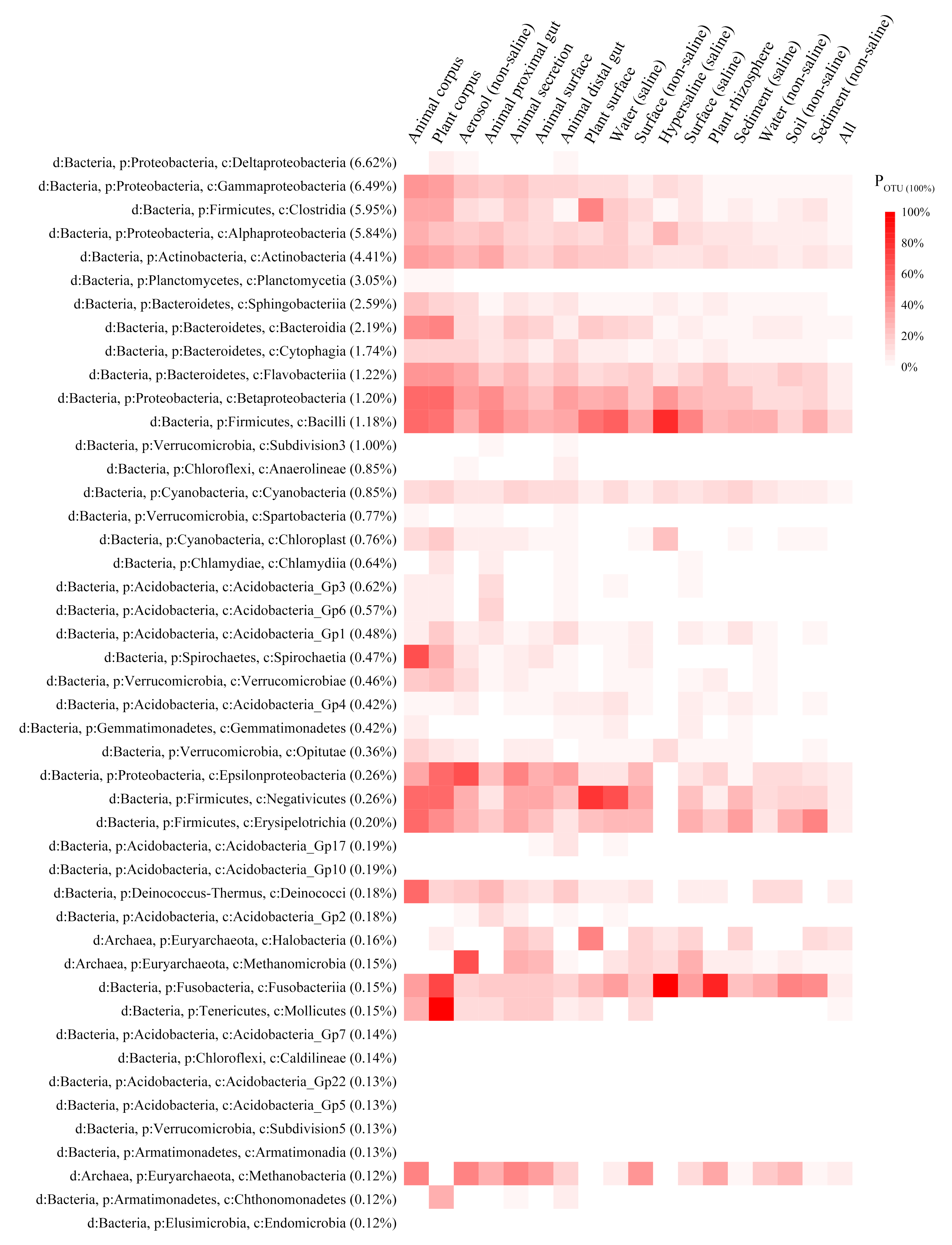

Supplement: Supplementary file 13 — Additional file 12: Supplementary Fig. S12. Genome-sequenced proportion of 46 main classes of prokaryotic predominant taxa. The parenthesis shows the OTU number proportion of each class in all prokaryotic OTUs. [file 40168_2020_903_MOESM12_ESM.tif]

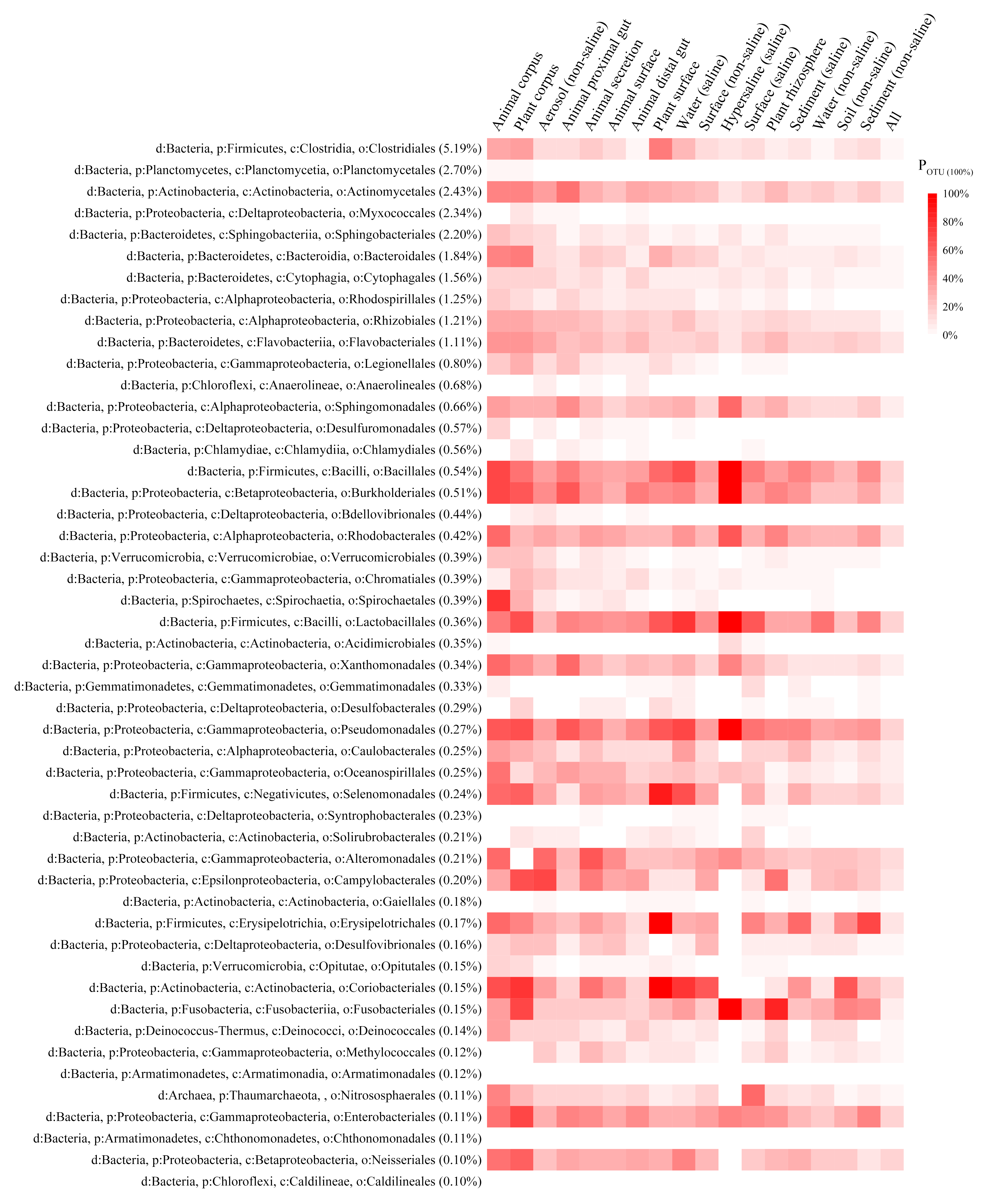

Supplement: Supplementary file 14 — Additional file 13: Supplementary Fig. S13. Genome-sequenced proportion of the 49 main orders of prokaryotic predominant taxa. The parenthesis shows the OTU number proportion of each order in all prokaryotic OTUs. [file 40168_2020_903_MOESM13_ESM.tif]

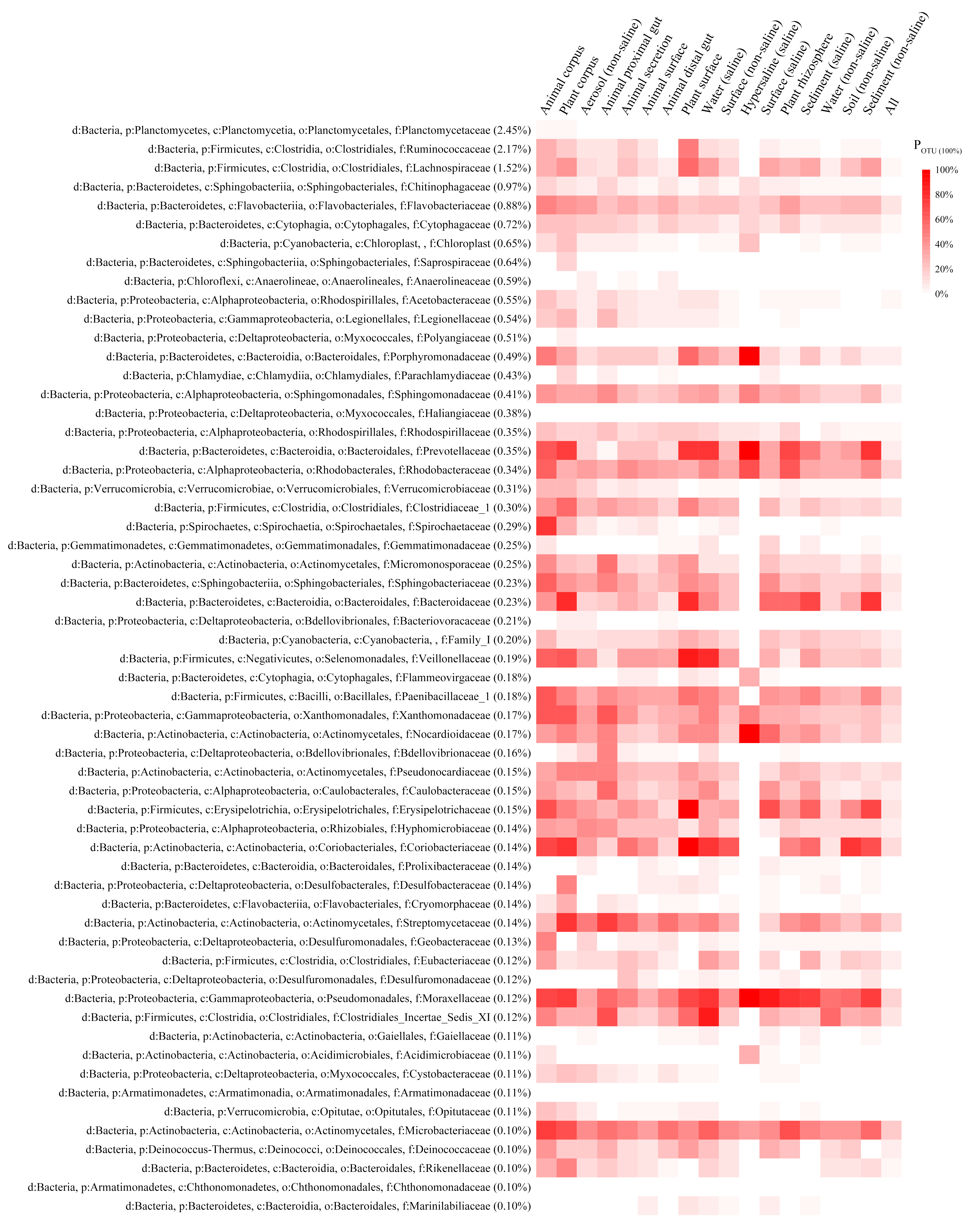

Supplement: Supplementary file 15 — Additional file 14: Supplementary Fig. S14. Genome-sequenced proportion of the 58 main families of prokaryotic predominant taxa. The parenthesis shows the OTU number proportion of each family in all prokaryotic OTUs. [file 40168_2020_903_MOESM14_ESM.tif]

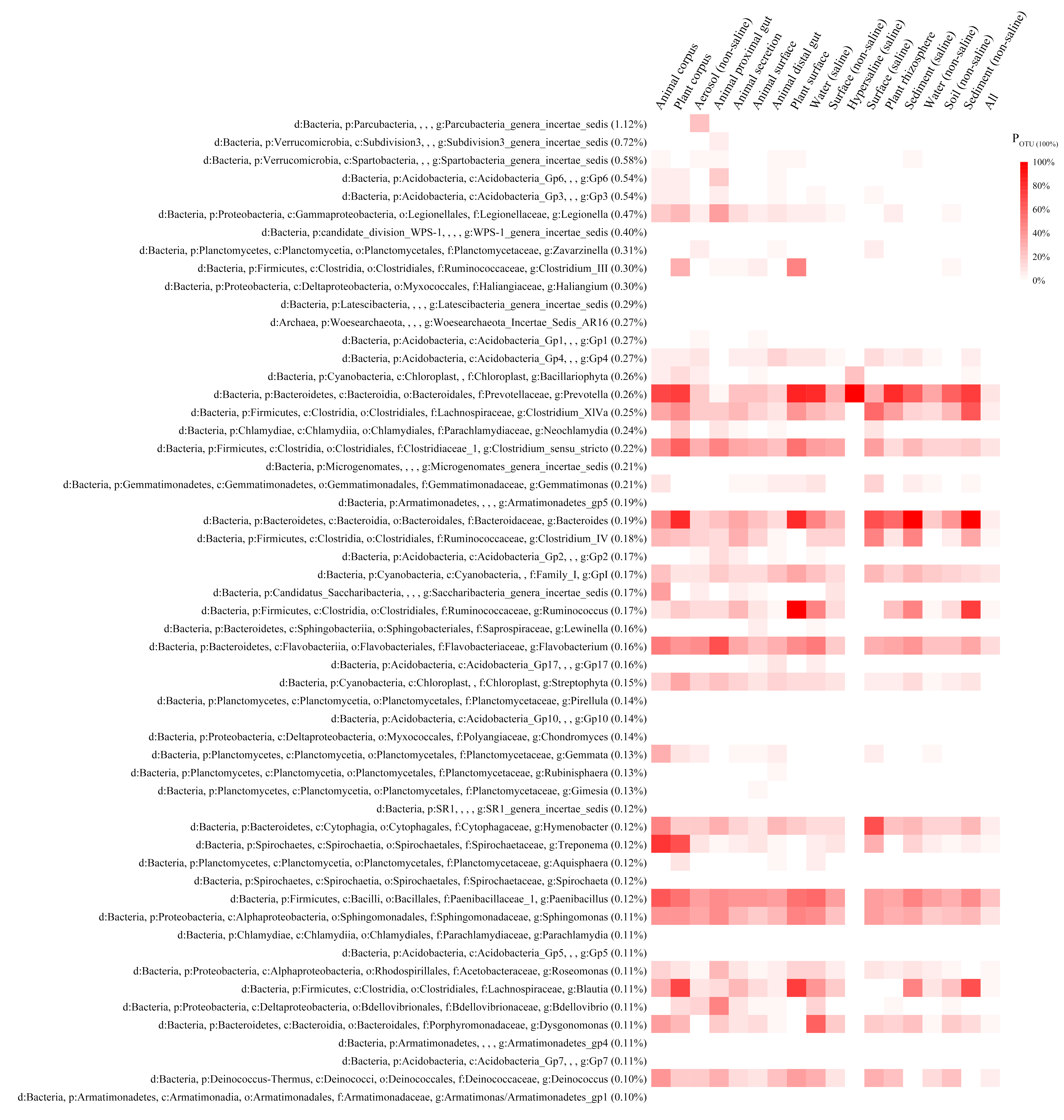

Supplement: Supplementary file 16 — Additional file 15: Supplementary Fig. S15. Genome-sequenced proportion of 55 main genera of prokaryotic predominant taxa. The parenthesis shows the OTU number proportion of each genus in all prokaryotic OTUs. [file 40168_2020_903_MOESM15_ESM.tif]

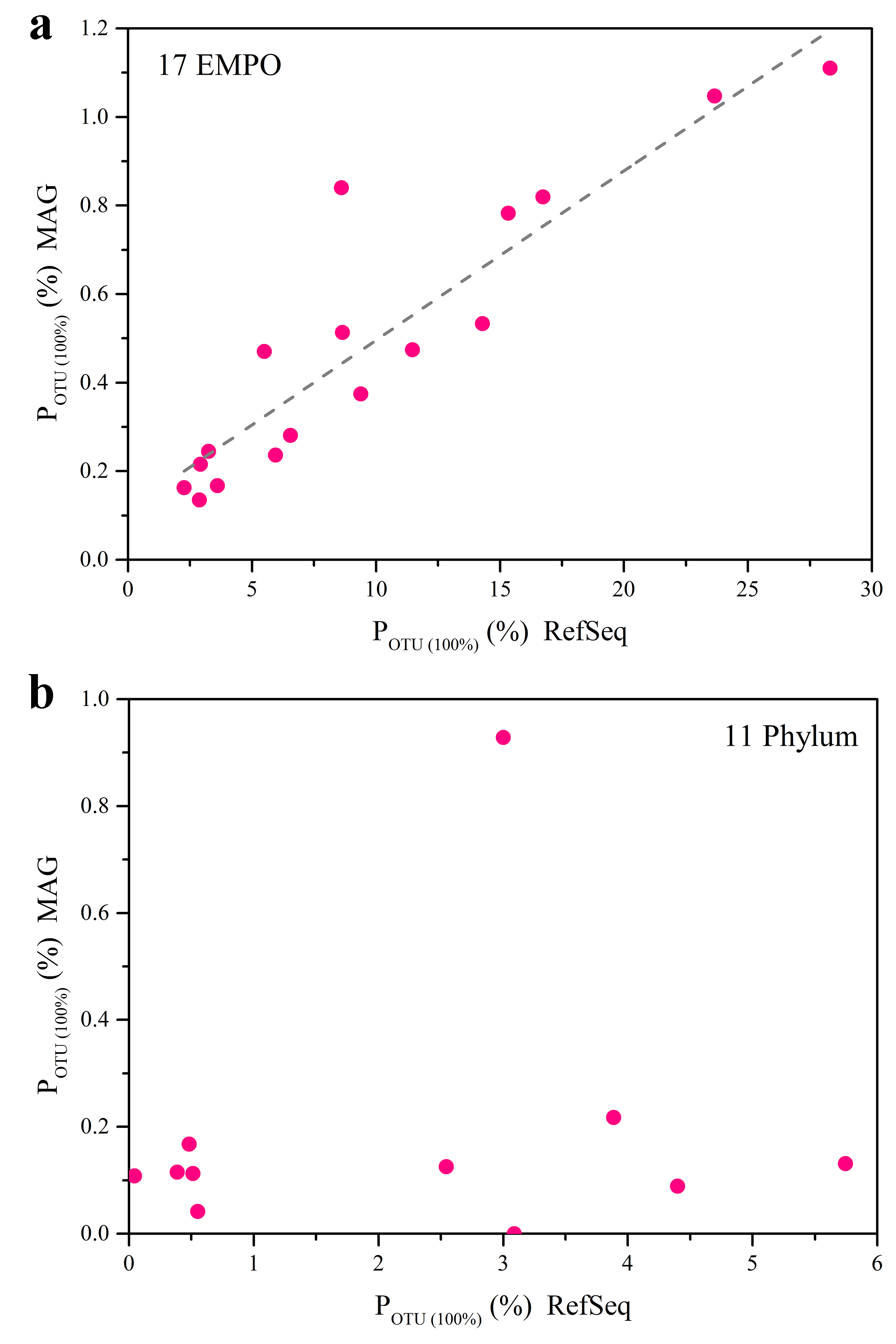

Supplement: Supplementary file 17 — Additional file 16: Supplementary Fig. S16. Culturability alters genome-sequenced preferences among prokaryotes but not environments. a, For the 17 environment types, the POTU (100%) based on MAGs is highly positively correlated with that based on RefSeq (r = 0.91, p < 0.01). b, For the 11 phyla with an OTU number proportion greater than 1%, the POTU (100%) based on the MAGs has no significant correlation with that based on the RefSeq (p > 0.05). [file 40168_2020_903_MOESM16_ESM.tif]
